# Aryl-quinoline-4-carbonyl hydrazone bearing different 2-methoxyphenoxyacetamides as potent α-glucosidase inhibitors; molecular dynamics, kinetic and structure–activity relationship studies

**DOI:** 10.1038/s41598-023-50395-8

**Published:** 2024-01-03

**Authors:** Haleh Hamedifar, Mahroo Mirfattahi, Minoo Khalili Ghomi, Homa Azizian, Aida Iraji, Milad Noori, Ali Moazzam, Navid Dastyafteh, Ali Nokhbehzaim, Katayoun Mehrpour, Shahrzad Javanshir, Somayeh Mojtabavi, Mohammad Ali Faramarzi, Bagher Larijani, Mir Hamed Hajimiri, Mohammad Mahdavi

**Affiliations:** 1 CinnaGen Medical Biotechnology Research Center, Alborz University of Medical Sciences, Karaj, Iran; 2 CinnaGen Research and Production Co., Alborz, Iran; 3 Endocrinology and Metabolism Research Center, Endocrinology and Metabolism Clinical Sciences Institute, Tehran University of Medical Sciences, Tehran, Iran; 4 Department of Medicinal Chemistry, School of Pharmacy, Iran University of Medical Sciences, Tehran, Iran; 5 Research Center for Traditional Medicine and History of Medicine, Department of Persian Medicine, School of Medicine, Shiraz University of Medical Sciences, Shiraz, 7134845794 Iran; 6 Stem Cells Technology Research Center, Shiraz University of Medical Sciences, Shiraz, Iran; 7 Pharmaceutical and Heterocyclic Chemistry Research Laboratory, Department of Chemistry, Iran University of Science and Technology, Tehran, 16846-13114 Iran; 8 Student Research Committee, Alborz University of Medical Sciences, Karaj, Iran; 9 Department of Pharmaceutical Biotechnology, Faculty of Pharmacy and Biotechnology Research Center, Tehran University of Medical Sciences, Tehran, Iran; 10 Nano Alvand Company, Avicenna Tech Park, Tehran University of Medical Sciences, Tehran, 1439955991 Iran

**Keywords:** Chemical biology, Drug discovery

## Abstract

Regarding the important role of α-glucosidase enzyme in the management of type 2 diabetes mellitus, the current study was established to design and synthesize aryl-quinoline-4-carbonyl hydrazone bearing different 2-methoxyphenoxyacetamide (**11a**–**o**) and the structure of all derivatives was confirmed through various techniques including IR, ^1^H-NMR, ^13^C-NMR and elemental analysis. Next, the α-glucosidase inhibitory potentials of all derivatives were evaluated, and all compounds displayed potent inhibition with IC_50_ values in the range of 26.0 ± 0.8–459.8 ± 1.5 µM as compared to acarbose used as control, except **11f** and **11l**. Additionally, in silico-induced fit docking and molecular dynamics studies were performed to further investigate the interaction, orientation, and conformation of the newly synthesized compounds over the active site of α-glucosidase.

## Introduction

Nowadays, diabetes categorizes as the 6th cause of global mortality, and among different types of diabetes, type 2 diabetes mellitus (T2DM) accounts for 90% of the diabetic population, which has become a major health problem worldwide^1,2^. Insulin is the hormone secreted by pancreatic β-cells and maintains glucose homeostasis by binding to cell receptors to transfer glucose. Also, insulin stimulates cells to get glucose from the blood and help the liver with glucose metabolism to lower glucose level to the normal condition^3^. Regarding the pathophysiology of T2DM, deficient insulin secretion by β-cells, tissue insulin resistance, and an inadequate compensatory insulin secretory response leads to high blood glucose level. This, in return associated with severe complications like, neuropathy, retinopathy, nephropathy, and cardiovascular problems^4^.

α-glucosidase (EC 3.2.1.20) is among the hydrolase group that attracted much attention due to its identification as a candidate for regulating blood glucose levels^5^. α-glucosidase hydrolyze 1,4-α-glucopyranosidic of oligosaccharide and disaccharide to produce monosaccharides contributing to the increase of glucose level. Acarbose, miglitol, and voglibose are commercially available α-glucosidase inhibitors; however, the routine consumption of these drugs is associated with bloating, diarrhea, and flatulence^6^. As a result, developing new α-glucosidase inhibitors is a valuable approach that slows down the catalytic activity of carbohydrates digestive enzyme^7^.

**Figure 1:**
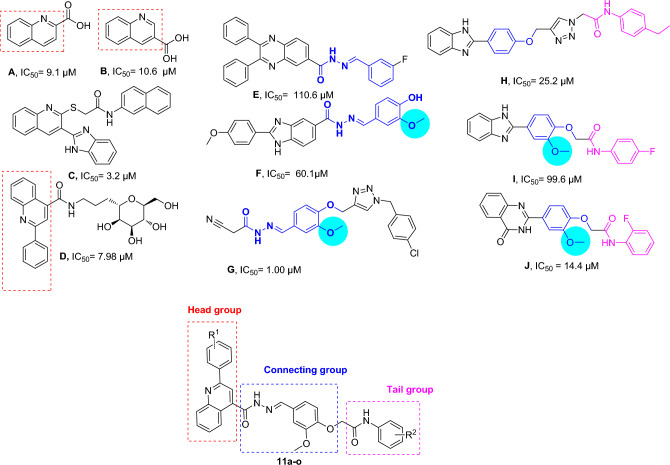
Demonstration of previously reported α-glucosidase inhibitors and designed compound.

Heterocyclic compounds are an undeniable part of drug design, and amongst quinoline, as a heterocycle ring exhibited various pharmacological activities^8^. Also, functionalized quinoline moieties are essential pharmacophoric with various therapeutic properties as antimicrobial, anticancer, anti-inflammatory, antioxidant, and antidiabetic agents^8,9^. The primary structure of quinoline (compounds **A** and **B**, Fig. 1) revealed good activity against α-glucosidase compared with the positive control acarbose (IC_50_ = 66.5 ± 1.5 µg/mL). In previous work, we identified that introducing electron-donating groups and specious substitutions on compound **C** enhanced the inhibitory enzyme potential^10^. In another study, it was shown that compound **D** had higher inhibitory activity than positive control and exhibited selective and effective photodegrading ability against an α-glucosidase target upon photo-irradiation^11^.

Schiff base derivatives are also present in some potent α-glucosidase inhibitors in these cases, compounds **E**, **F,** and **G**^12^ are noble examples. The analysis of these derivatives reveals that this connecting group is critical for the interactions with the binding site of the enzyme and, consequently enzyme inhibition. The same is also evident in our previously reported series of cyanoacetohydrazide linked to 1,2,3-triazoles where the acetohydrazide linker is indeed a key structural feature governing their anti-α-glucosidase potency. Also, evaluation of the type of substitution on the phenoxy linker exhibited that methoxy substitution on the phenoxy linker significantly increased the activity^13^.

Arylacetamide derivatives have been developed and investigated as anti-diabetic agents. Diphenylimidazole core attached to the various N-aryl acetamides (Fig. 1, Compound **H**) were developed as α-glucosidase inhibitors with IC_50_ values of 25.2 to 176.5 μM compared with the standard inhibitor acarbose (IC_50_ = 750.0 μM). Compound **H** exhibited a competitive mode of inhibition with *K*_*i*_ = 23 μM against α-glucosidase. In silico studies showed that the phenoxy linker of potent inhibitor exhibited H-bound interaction with Asp616 and/or Asp282^14,15^. Also, compound **I** with benzimidazole-phenoxyacetamide structure was a competitive inhibitor of α-glucosidase with *K*_*i*_ of 67.0 μM with 45.0 ± 0.8% α-amylase inhibition at 108 μM^16^. In 2022, quinazolin-4(3H)-one linked to phenoxy-acetamide derivatives (**J**) was synthesized and exhibited IC_50_ in the range of 14.4 ± 0.2 µM to > 750 µM was observed. In silico study showed phenoxy acetamide participated in several H-bound as well as hydrophobic interactions with Asp616, Arg600, and Trp48 of the enzyme active site^17^. Arylacetamide moiety seems to provide a suitable site for derivitization and proved to possess α-glucosidase inhibition.

Using a rational design of new molecules, we have chosen aryl-quinoline-4-carbonyl hydrazone conjugated to different 2-methoxyphenoxyacetamide moieties. The structure of all derivatives was confirmed with IR, ^1^H-NMR, ^13^C-NMR, and elemental analysis. The synthesized derivatives were evaluated as α-glucosidase inhibitors, and the kinetic study of the most active analog was performed. Also, molecular dynamic studies of the most potent derivative were executed.

## Results and discussion

### Chemistry

**Scheme 1:**
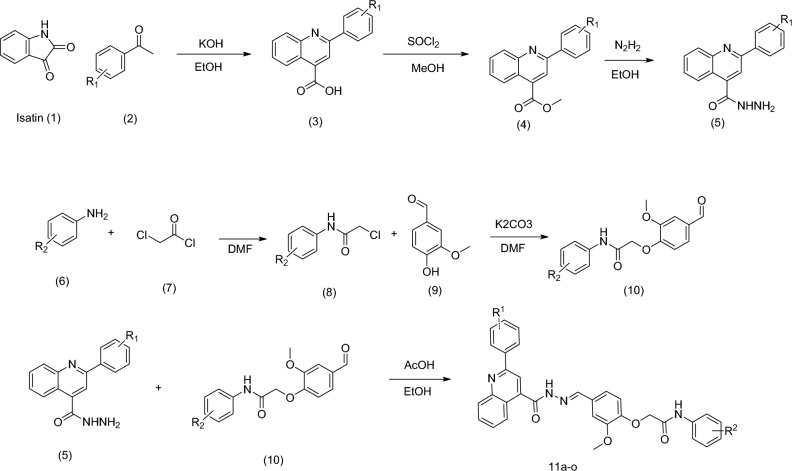
Synthesis of the final compounds.

The synthesis of new aryl-quinoline-4-carbonyl hydrazone bearing different 2-methoxyphenoxyacetamide derivatives is presented in Scheme 1. Starting with commercially available isatin (**1**), substitutions with various commercially available acetophenone derivates (**2**) afforded intermediaries (**3**). Additionally, 2-Aryl-quinoline-4-carboxylic acid (**3**) was suspended in SOCl_2_ and after the 2 h, refluxed, and the mixture was cooled to 0 °C. Methanol was added to get the hydrochloride salt of methyl ester derivative (**4**). Subsequently, hydrazine hydrate in ethanol was added, and the mixture was refluxed to afford **5** derivatives. In the other reaction, aniline derivatives (**6**) were added to chloroacetylchloride (**7**) to get the. Next, 4-hydro-3-methoxy benzaldehyde was charged with 2-chloro-N-phenyl acetamide derivatives (**8**), followed by the addition of potassium carbonate to afford **10**. Finally, hydrazide derivatives (**5**) were treated with the 2-(4-formyl-2-methoxyphenoxy)-N-aryl-acetamide derivatives (**10**) to provide the final products **11a**–**o**. Synthesis details are given in the experimental section.

The NMR results confirmed the structures of all synthesized compounds, for example, in ^1^H-NMR, individual peaks of the H group of –NH–N= appear in ~ 12 ppm, the H of NH group appears in ~ 10 ppm, H of N=CH appears in ~ 8.5 ppm. Also, the H of acetamide appears in around 5 ppm confirming the production of final products.

### Biological evaluation and structure–activity relationships

The focused library of optimized **11a–o** and their inhibitory potencies against α-glucosidase enzyme are presented in Table 1. To better evaluate the SARs, these analogs were divided into two categories based on the type of moiety at the R^1^ position, **11a–g** (R^1^ = H), **11h–o** (R^1^ = OCH_3_).

**Table 1:** The α-glucosidase inhibition assay of **11a**–**o**.


Compounds	R^1^	R^2^	IC_50_ (µM)	Concentrations of precipitation (µM)
**11a**	H	2-CH_3_	34.8 ± 1.1	–
**11b**	H	4-CH_3_	95.2 ± 1.2	≥ 375
**11c**	H	4-OCH_3_	43.7 ± 1.0	–
**11d**	H	4-CH_2_-CH_3_	156.8 ± 1.5	≥ 375
**11e**	H	4-F	52.6 ± 0.6	–
**11f**	H	4-Cl	> 750	≥ 375
**11g**	H	4-Br	180.9 ± 1.7	≥ 375
**11h**	OCH_3_	H	62.4 ± 0.9	≥ 375
**11i**	OCH_3_	2-CH_3_	71.2 ± 0.6	–
**11j**	OCH_3_	4-OCH_3_	61.8 ± 0.5	≥ 190
**11k**	OCH_3_	4-CH_2_-CH_3_	26.0 ± 0.8	–
**11l**	OCH_3_	4-F	> 750	≥ 190
**11m**	OCH_3_	4-Br	152.2 ± 1.7	≥ 190
**11n**	OCH_3_	4-Cl	48.7 ± 0.9	≥ 190
**11o**	OCH_3_	4-NO_2_	459.8 ± 1.5	–
**Acarbose**	–	–	750.0 ± 2.0	–

Data represented in terms of mean ± SD.

In the case of **11a–h**, the methyl-containing derivatives (**11a** and **11b**) improved the inhibition compared to the positive control, and the *ortho* position exhibited better potency *vs para* analog. The replacement of 4-methyl with a 4-methoxy moiety (**11c**) in general enhanced the potency compared with their methyl counterparts. Similarly, the *para* position recorded lower potency. Furthermore, an inhibitory effect of **11d** was found to be IC_50_ = 156.8 µM, which contained a 4-ethyl substituent. It was understood that the increased bulkiness at the *para* position is not tolerated. Also, in most, the compounds encompassing halogen substitution showed the deterioration of the enzyme inhibition, and the best results came back to fluorine with a smaller size and strong electron-withdrawing power, followed by the bromine group. Unexpectedly *para*-chlorine substitution completely lost its potency.

To get more insight into the SARs properties, derivatives **11h**–**o** were synthesized bearing OCH_3_ at R^1^ position. **11h** derivatives as the unsubstituted analog of methoxy series exhibited an IC_50_ value of 62.4 ± 0.9 µM, which is more than 11-fold more potent *vs* positive control. Subsequently, electron-donating groups on the phenyl ring were synthesized. **11i** bearing 2-CH_3_ slightly reduced the potency compared while 4-OCH_3_ substitution (**11j**) slightly improved the activity. Noteworthy replacement of methoxy moiety with 4-CH_2_-CH_3_ (**11k**) as bulk electron donating group resulting in the most potent derivative with IC_50_ of 26.0 µM. To test the electron-withdrawing properties at the R^2^ position, we also synthesized **11l**–**o** derivatives. The inhibitory capability of these derivatives recorded a reduction in the potency and even the NO_2_ moiety did not empower the activity in comparison with **11h** as an unsubstituted derivative. The exception in this trend came back to 4-Cl (**11n**) with an IC_50_ value of 48.7 µM.

In general, almost all of the reported compounds except **11f** and **11l** showed higher anti-α-glucosidase potency in comparison with the positive control. Also, a closer look at the results exhibited that series **11a**–**g** (R^1^ = H), and **11h**–**o** (R^1^ = OCH_3_) demonstrated opposite results. 4-ethyl substitution of methoxy analogs (compound **11k,** R^1^: OCH_3_, R^2^: 4-CH_2_-CH_3_; IC_50_ = 26.0 µM) was the most potent derivative, while such substitution (**11d**) in the R^1^ = H derivatives was one of the least active compounds. The same trend was seen between **11f** (R^1^: H, R^2^: 4-Cl; IC_50_ > 750 µM) *vs* **11n** (R^1^: 4-OCH_3_, R^2^: 4-Cl; IC_50_ = 48.7 µM) as well as **11g** *vs* **11m**. This pinpoints that the type of substitution at the R^1^ position is an issue; however, the impact of R^2^ substitution seems to be even more significant, as already noted.

Overall, the intricate differences in inhibitory potencies among the synthesized compounds against α-glucosidase can be attributed to a combination of steric, electronic, and conformational effects introduced by the subtle substituent differences. A closer examination of the results revealed that the impact of R^2^ substitution seemed more significant than that of R^1^ substitution. Compound **11k**, with 4-ethyl substitution (R^1^: OCH_3_, R^2^: 4-CH_2_-CH_3_; IC_50_ = 26.0 µM), stood out as the most potent, emphasizing the importance of specific R^2^ substitutions. The contrasting results between compounds **11f** and **11l**, as well as **11g** and **11m**, highlight that the type of substitution at the R^1^ position plays a crucial role in the observed variations.

### Enzyme kinetic studies

**Figure 2:**
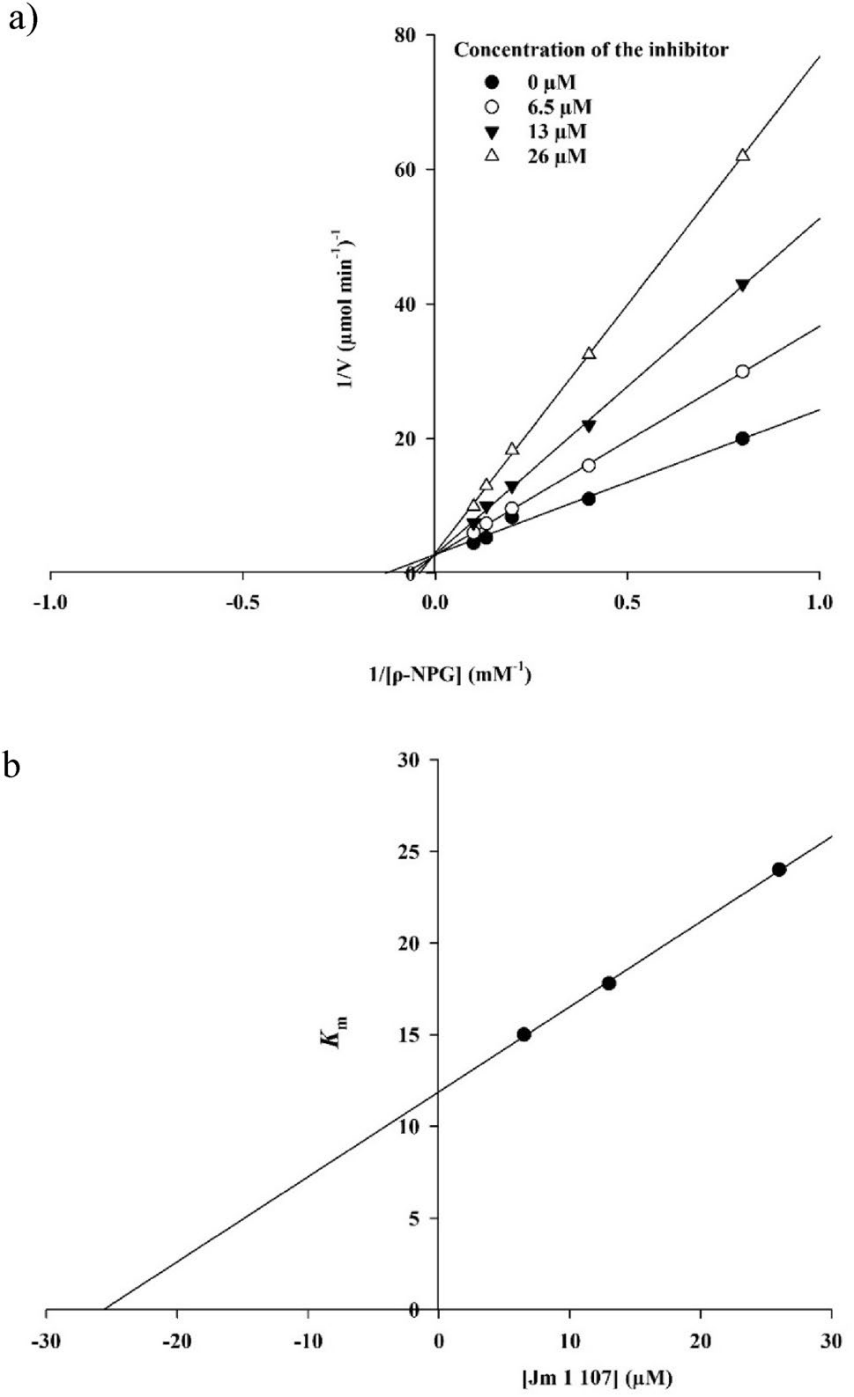
Kinetics of α-glucosidase inhibition by **11k**. (**a**) The Lineweaver–Burk plot in the absence and presence of different concentrations of **11k**; (**b**) The secondary plot between *K*_m_ and various concentrations of **11k**.

Based on the Lineweaver–Burk plot shown in Fig. 2a, it can be observed that the *K*_*m*_ (Michaelis–Menten constant) gradually increased while the *V*_*max*_ (maximum reaction rate) remained unchanged with increasing inhibitor concentration. This pattern indicates a competitive inhibition mechanism. The results suggest that compound **11k** binds to the enzyme's active site and competes with the substrate for binding. Additionally, by plotting the *K*_*m*_ values against different inhibitor concentrations, an estimation of the inhibition constant (*K*_*i*_) was obtained. The analysis yielded a *K*_*i*_ value of 25.6 µM, as depicted in Fig. 2b.

### Docking study

A validated molecular docking procedure based on our previous studies was used to assess the binding mode of newly synthesized compounds **11a**–**o** and compare them with acarbose as the standard inhibitor of α-glucosidase. The reliability of the docking method was established by redocking the α-glucosidase natural substrate conducted by the previous study^18^.

Through previous investigation, the α-glucosidase active site is formed at the interface of domain A and domain B in which the residues from each side contribute to the active site region. In this way, phenylalanine residues play an important role at the entrance part of the active site. Furthermore, an active site lid is made up of residues 308–313, which plays an important role in substrate movement into the active site. Additionally, there are three conserved catalytic residues inside the active site pocket. They include Asp214 (nucleophile), Glu276 (general acid/base), and Asp 349, which stabilizes the substrate at the active site pocket^19^.

**Figure 3:**
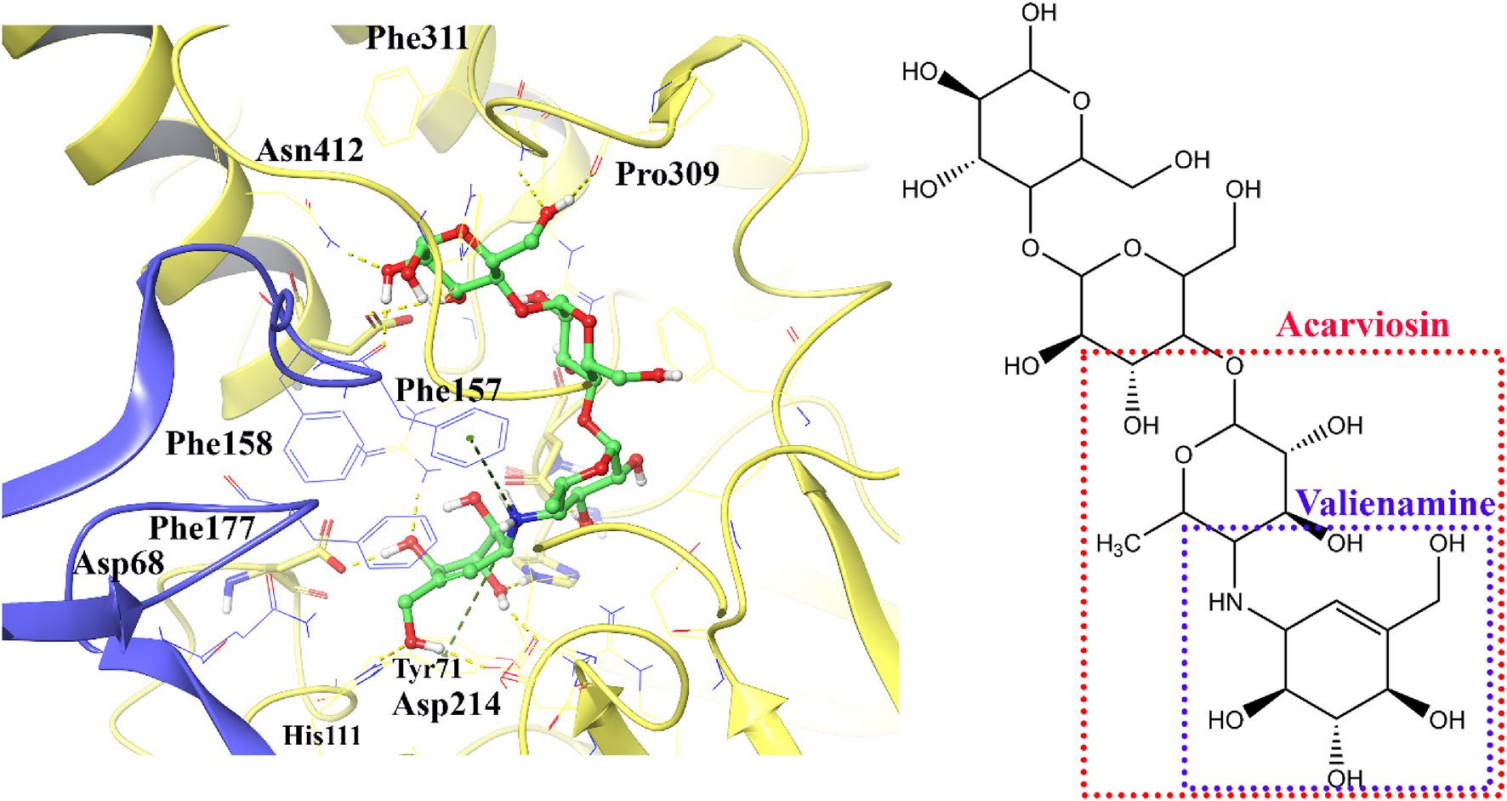
3D representation of acarbose interaction at the α-glucosidase active site. The A domain of the α-glucosidase is depicted in yellow, and the B domain is shown in blue.

Figure 3 shows the interactions of acarbose in which the non-reducing end of acarbose (acarviosine moiety) is responsible for many important interactions with the enzyme. The valienamine moiety has hydrogen bonds to many residues in the − 1 subsite, including; the conserved catalytic nucleophile, Asp214, and other residues like Asp68, Arg439, and His111. The protonated amino group interacts with Phe157 at the B domain through π-cation interaction. Also, at subsite, + 1 acarviosine interacts with the conserved Asp349 through hydrogen bonds. By contrast, the reducing end of acarbose has several interactions with residues at the subsites + 2 and + 3 residues, including; Phe157, His239, Pro309, Arg312, Asp408, and Asn412.

**Figure 4:**
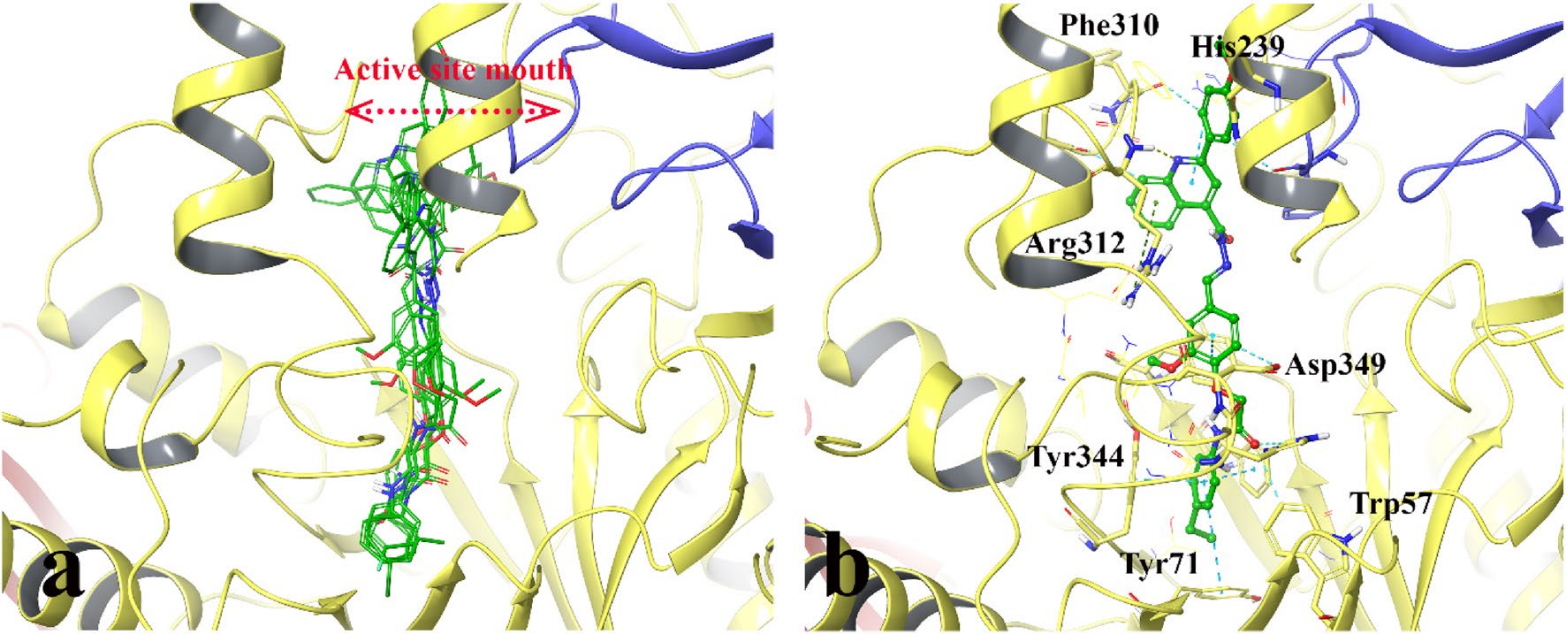
3D poses of the docked structure of the synthesized compound over the α-glucosidase active site. The superimpose structure (**a**) and the IFD pose of the most promising compound, **11k** (**b**). The A domain of the α-glucosidase is depicted in yellow, and the B domain is shown in blue.

Figure 4a depicts the superimposed conformations and orientations of some of the synthesized compounds. It indicates that these compounds overall have similar conformations in which the 4-substituted acetanilide part of compounds oriented toward − 1 subsite is in the same position as the non-reducing end of acarbose (acarviosin moiety). Also, the quinoline nucleus provided the same orientation as the reducing part of acarbose through interaction at the active site mouth of the enzyme. Furthermore, Fig. 4b shows the non-bonding interactions of compound **11k**, as the best experimental active compound. The aromatic ring in the acetanilid moiety of the mentioned compound makes two π-π interactions with Phe298 and Tyr344. The aromatic ring in the dimethoxy benzene moiety makes a π-π interaction with Phe300 and an aromatic-hydrogen bond interaction with the conserved catalytic residue Asp349. Furthermore, the phenyl quinoline group interacts with Phe157, Ser308, Phe310, Arg312, and Asn412. The residues from 308 to 313 form the active site lid that plays an important role in substrate movement into the active site.

### Molecular dynamic (MD) investigation

**Figure 5:**
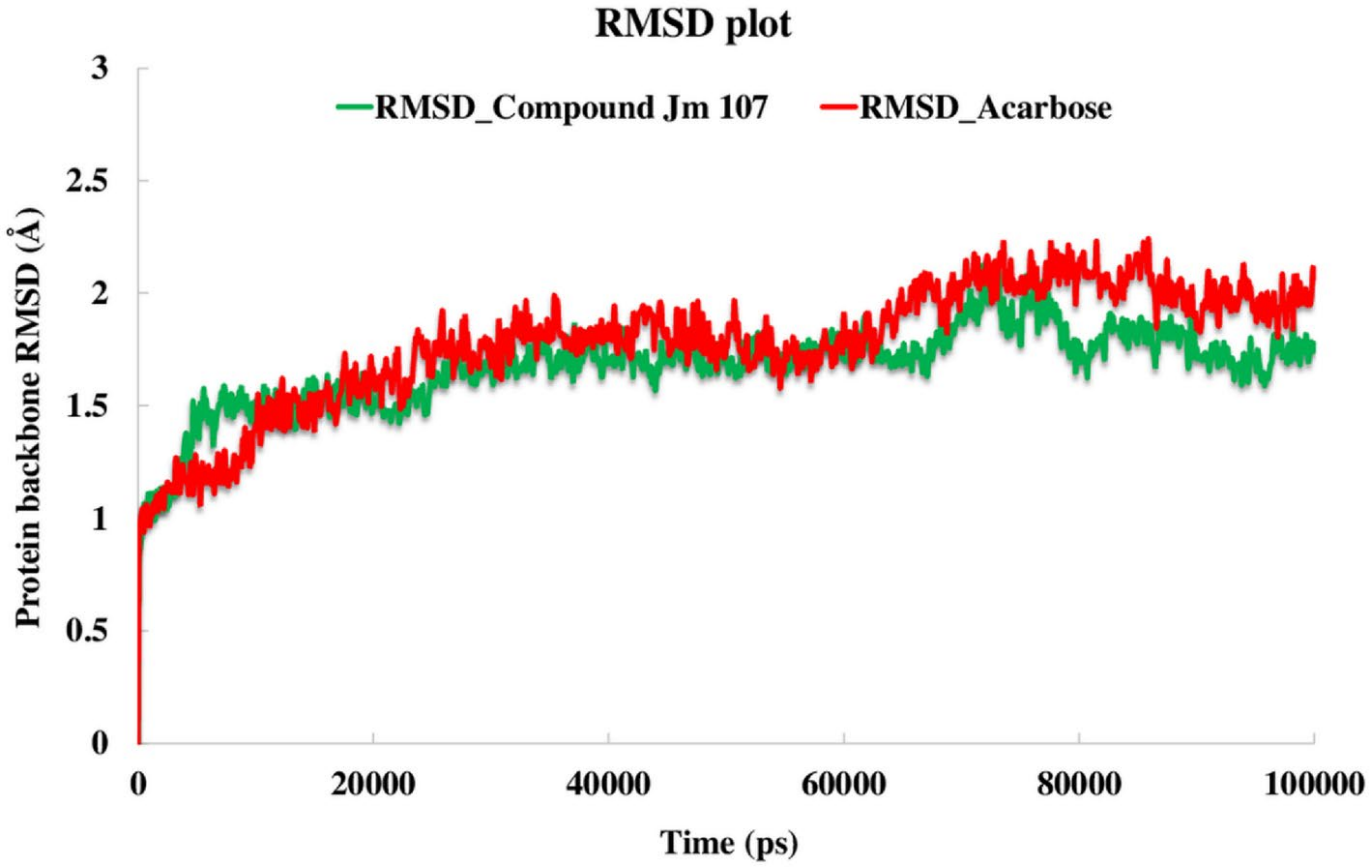
RMSD plot of α-glucosidase backbone in complex with acarbose (red) and compound **11k** (green), throughout the 100 ns of the simulation time.

To study the stability of the dynamic behavior of the system, the best IFD pose of compound **11k** and acarbose were implemented as starting points for 100 ns MD simulation to predict the motion of complex systems at an atomistic level^20^. Root mean square deviation (RMSD) values are indicative of the conformational stability and perturbations of the system. When RMSD values no longer follow a specific trend but fluctuate around a certain point, it can be argued that the complex has reached equilibrium^21^. Figure 5 depicts the protein backbone RMSD values for the α-glucosidase-**11k** complex (in green) and α-glucosidase-acarbose complex (in blue) over about 100 ns MD simulation time. The RMSD value of the α-glycosidase complexed with compound **11k** shows the same value as the enzyme complex with acarbose at the first 60 ns. Also, for the rest of the simulation time, the RMSDs plot became slightly increased and differentiative for both complexed systems in which it became a little lower for α-glucosidase-**11k** complex than acarbose one (about 1.75 Å and 2.1 Å, respectively). The such observation indicated that the employed simulation time was enough to obtain an equilibrium structure over the simulation time.

**Figure 6:**
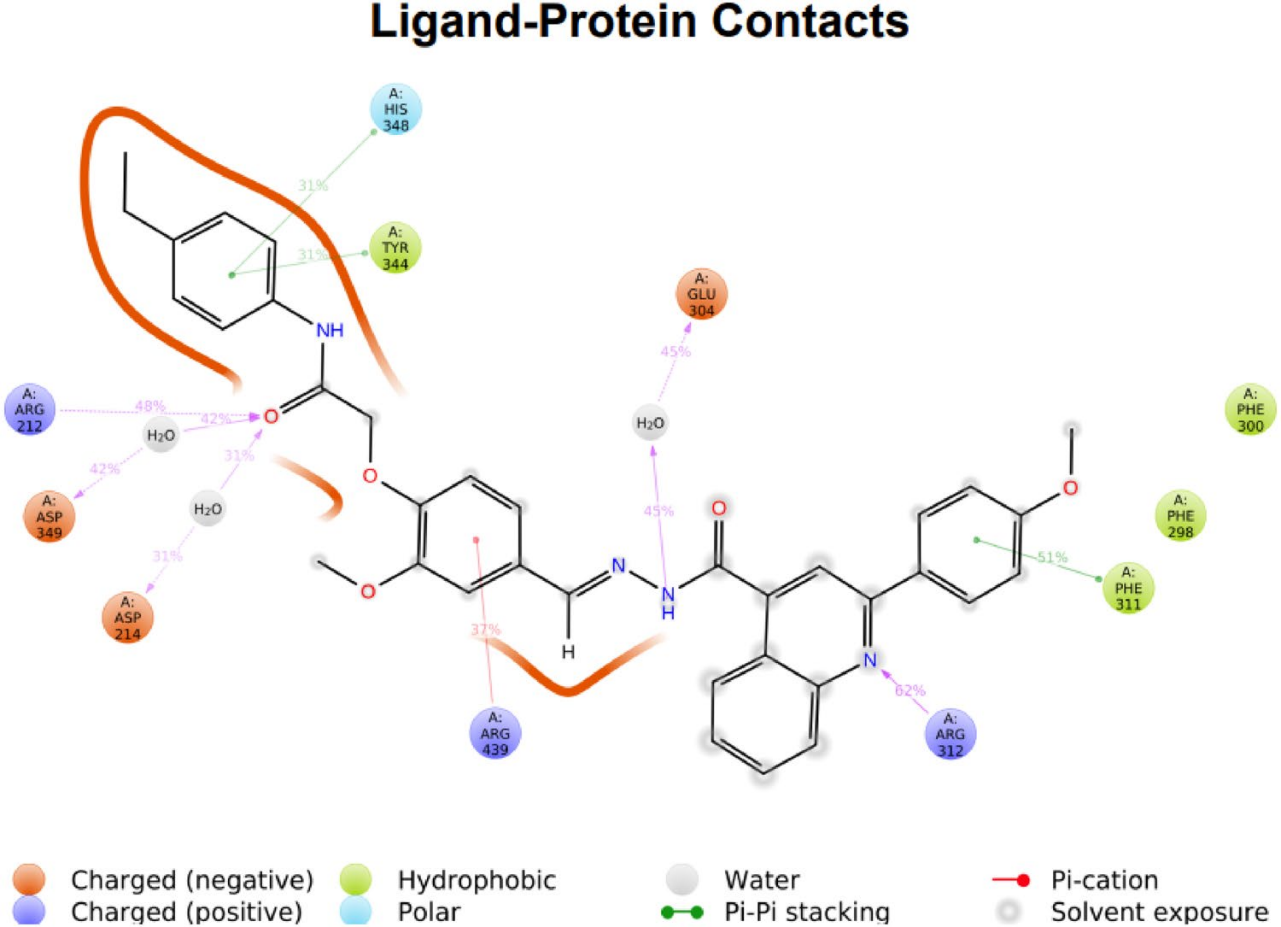
2D interaction diagram of compound **11k**—α-glucosidase complex which is responsible for over 30% of MD simulation time.

Furthermore, the 2D interactions diagram of compound **11k** in complex with the enzyme is depicted in Fig. 6. These interactions happened at least 30% duration of the MD simulation time. Figure 6 shows that the carbonyl oxygen of acetanilide moiety interacted with catalytic residues of Asp349 and Asp214 through water-mediated hydrogen bonds for about 42% and 39% of the simulation time, respectively. Also, at this part, the phenyl group stabilized through hydrophobic π–π stacking non-bonding interaction by His 348 and Tyr344 residues for about 30% of MD simulation time.

Also, the 2-methoxy phenyl quinoline moiety stabilized at the active site opening space through non-bonding interaction with Phe311 and Arg 312 for over half of the MD simulation time (51% and 62%, respectively). Moreover, the NH group belonging to the hydrazone provides water-mediated H-bond interaction with Glu304 for about 45% of MD simulation time. It is proved that the pose of compound **11k,** which resulted by the IFD investigation, is stable in that the acetanilide moiety is deeply inserted inside the active site pocket while the phenyl quinoline part is oriented between the entrance of the active site pocket.

In summary, through MD simulation investigation, it is revealed that the resulted in IFD posed of compound **11k** is stable at the α-glucosidase active pocket in which the 4-ethyl acetanilide part of compound **11k** oriented in − 1 subsite which is in the same position as the non-reducing end of acarbose (acarviosine moiety) and is responsible for many important interactions through catalytic residue. Also, the 2-methoxy quinoline part provided the same orientation as the reducing part of acarbose through interaction at the active site mouth of the enzyme.

## Conclusion

We describe the design and synthesis of aryl-quinoline-4-carbonyl hydrazone bearing different 2-methoxyphenoxyacetamide compounds, and we have assessed the inhibitory effects of all compounds against α-glucosidase through in vitro assays. The results revealed that **11k** incorporating OCH_3_ at R^1^ and 4-CH_2_-CH_3_ at R^2^ displayed the best inhibition potency against a-glucosidase with an IC_50_ value of 26.0 ± 0.8 μM. The SAR outcomes highlighted that the type of substitution at the R^1^ position of phenyl-quinoline is important; however, the impact of R^2^ substitution on acetamide seems to be even more significant effect, as already noted. Additionally, the kinetic investigation of **11k** as the most potent derivative offered the competitive type of α-glucosidase. Finally, an in silico study revealed that **11k** interacted with Glu304, Phe311, Arg312, Tyr344, His348, and Arg439 of the active site. These findings will be prominent for designing α-glucosidase inhibitors in drug discovery.

## Experimental

### General

All the reagents were purchased from commercial sources. ^1^H and ^13^C NMR spectra were determined by a Bruker Advance spectrometer 400 MHz spectrometer. All the chemical shifts were reported as (*δ*) values ppm. Multiplicities were indicated by s (singlet), d (doublet), t (triplet), q (quartet), m (multiplet), and coupling constant *J* was reported in hertz (Hz). CHN analysis was performed using Costech Company. IR spectra were obtained with a Nicolet, FR-IR Magna 550. Melting points were also recorded using Kofler hot-stage apparatus. All the chemicals were purchased from Merck, Germany, and Sigma, Germany.

### Synthesis

#### Synthesis of 2-aryl-quinoline-4-carboxylic acid derivatives (**3**)

Isatin (**1**, 13.66 mmol) was suspended in 55 mL of ethanol and heated to 65 °C to dissolve the solid. A solution of potassium hydroxide (33% w/v, 6.7 mL) was added and stirred at 65 °C for 15 min. Acetophenone derivates (**2**, 15.4 mmol) were added dropwise to the heated solution. Upon complete addition, the reaction solution was warmed to reflux. After 48 h, the reaction solution was concentrated to a dark brown solid. A solution (60 mL) of 20% acetic acid in water was added slowly to adjust the pH to 5. The precipitate that formed was vacuum filtered and washed with EtOH and hexanes.

#### Synthesis of methyl 2-Aryl-quinoline-4-carboxylate derivatives (**4**)

2-Aryl-quinoline-4-carboxylic acid (**3**, 10 mmol) was suspended in SOCl_2_ (12 mmol) at 0 °C. The mixture was refluxed for 2 h and cooled to 0 °C. Methanol (5 mL) was added to get the hydrochloride salt of the methyl ester derivative. Cold water (15 mL) was added to the mixture and its pH was adjusted to 7.0 with saturated with aqueous NaHCO_3_ solution. The precipitated solid was filtered, washed with cold water (2 × 10 mL), and dried under reduced pressure to get 2-(phenyl derivates)-quinoline-4-carboxylic acid methyl ester.

#### Synthesis of methyl 2-Aryl-quinoline-4-carbohydrazide derivatives (**5**)

A mixture of methyl 2-arylquinoline-4-carboxylate and hydrazine hydrate (1 mL, 99%.) in ethanol (10–15 mL) was refluxed for 10–14 h. After completion of the reaction, the reaction mixture was cooled to room temperature and poured into crushed ice. The white solid thus obtained was filtered, washed with water, and recrystallized from ethanol.

#### Synthesis of 2-chloro-N-aryl-acetamide derivatives (**8**)

Aniline derivatives (**6**, 1 mmol) were dissolved in DMF (4 mL), and then chloroacetylchloride (**7**, 1.2 mmol) was added at 0 °C. At room temperature, the mixture was stirred for 5 h, and subsequently poured into water, followed by filtration to isolate the desired product. The resulting solids were filtered, dried, and recrystallized from ethanol.

#### Synthesis of 2-(4-formyl-2-methoxyphenoxy)-N- aryl-acetamide derivatives (**10**)

To a solution of 4-hydro-3-methoxybenzaldehyde (9, 1 mmol) in DMF (5 mL), 2-chloro-N-phenylacetamide derivatives (8, 1.1 mmol) were added, followed by the addition of potassium carbonate (1.2 mmol). After stirring at room temperature for 5 h, the reaction mixture was poured into 25 mL of ice water. The resulting product was collected by filtration and rinsed with water.

#### Synthesis of **11a–o** derivatives

4-substituted aldehyde (**10**, 1.1 mmol) and methyl 2-Aryl-quinoline-4-carbohydrazide derivatives (**5,** 1 mmol) and a few drops of glacial acetic acid as a catalyst in the absolute ethanol were refluxed for 6–7 h. The precipitate formed during the cooling of the mixture was filtered off, and washed two or three times with cold ethanol. Finally, the solid was recrystallized from ethanol (Supplementary Information).

##### 2-(2-methoxy-4-((2-(2-phenylquinoline-4-carbonyl)hydrazineylidene)methyl)phenoxy)-N-(o-tolyl)acetamide (**11a**)

Yield: 84%. Brown solid. M.p. > 250 °C. IR (KBr): 3422, 3105, 1676, 1558, 1342, 1175, 985 cm^−1^. TLC (40:60; Hexane/AcOEt); Rf = 0.48.^1^H NMR (400 M*Hz*, DMSO-*d*_6_) δ 12.21 (s, 1H, –NH–N =), 10.08 (s, 1H, NH), 8.39 (s, 1H, H_Ar_), 8.38–8.35 (m, 2H, H_Ar_), 8.34 (s, 1H, H_Ar_), 8.25 (d, *J* = 8.8 *Hz*, 2H, H_Ar_), 8.19 (d, *J* = 8.4 *Hz*, 1H, H_Ar_), 7.87 (d, *J* = 7.8 *Hz*, 1H, H_Ar_), 7.69 (t, *J* = 7.6 *Hz*, 1H, H_Ar_), 7.59 (d, *J* = 7.6 *Hz*, 2H, H_Ar_), 7.57–7.51 (m, 4H, H_Ar_), 7.53–7.45 (m, 1H, H_Ar_), 7.26 (d, *J* = 8.4 *Hz*, 1H, H_Ar_), 7.14 (d, *J* = 8.3 *Hz*, 2H, H_Ar_), 7.06 (d, *J* = 8.3 *Hz*, 1H, H_Ar_), 4.79 (s, 2H,OCH_2_), 3.92 (s, 3H,OCH_3_), 2.26 (s, 3H,CH_3_). ^13^C NMR (101 M*Hz*, DMSO-*d*_*6*_) δ 166.51, 163.18, 156.26, 150.12, 149.88, 149.25, 148.42, 141.90, 138.56, 136.37, 133.13, 130.85, 130.47, 130.14, 129.66, 129.42, 128.15, 127.82, 127.66, 125.63, 123.98, 122.49, 119.94, 117.72, 113.95, 109.27, 68.52, 56.09, 20.93. SI–MS (C_33_H_28_N_4_O_4_): calculated m/z 544.21 [M+H]^+^, observed m/z 544.32 [M+H]^+^; Anal. Calcd for C_33_H_28_N_4_O_4_: C, 72.78; H, 5.18; N, 10.29; Found: C 73.09; H 5.22; N 10.12.

##### 2-(2-methoxy-4-((2-(2-phenylquinoline-4-carbonyl)hydrazono)methyl)phenoxy)-N-(p-tolyl)acetamide (**11b**)

Yield: 81%. Brown solid. M.p. > 250 °C. IR (KBr): 3436, 3024, 1652, 1601, 1349, 1188 cm^−1^. TLC (40:60; Hexane/AcOEt); Rf = 0.44.^1^H NMR (400 M*Hz*, DMSO-*d*_6_) δ 12.21 (s, 1H, –NH–N=), 10.08 (s, 1H, NH), 8.39–8.34 (m, 4H, H_Ar_), 8.25 (d, *J* = 8.3 *Hz*, 1H, H_Ar_), 8.19 (d, *J* = 8.4 *Hz*, 1H, H_Ar_), 7.90–7.83 (m, 1H, H_Ar_), 7.72–7.66 (m, 1H, H_Ar_), 7.61–7.51 (m, 5H, H_Ar_), 7.49 (t,* J* = 8.1 *Hz*, 1H, H_Ar_), 7.26 (d, *J* = 8.4 *Hz*, 1H, H_Ar_), 7.14 (d, *J* = 8.1 *Hz*, 2H, H_Ar_), 7.06 (t, *J* = 8.3 *Hz*, 1H, H_Ar_), 4.79 (s, 2H, OCH_2_), 3.91 (s, 3H, OCH_3_), 2.26 (s, 3H,CH_3_).^13^C NMR (101 M*Hz*, DMSO-*d*_*6*_) δ 166.50, 163.17, 156.24, 150.10, 149.86, 149.24, 148.40, 141.88, 138.55, 136.36, 133.11, 130.83, 130.46, 130.13, 129.65, 129.40, 128.14, 127.90, 127.81, 125.62, 123.97, 122.48, 119.92, 117.71, 113.94, 109.26, 68.51, 56.09, 20.93. SI–MS (C_33_H_28_N_4_O_4_): calculated m/z 544.21 [M+H]^+^, observed m/z 544.27 [M+H]^+^; *Anal*. Calcd for C_33_H_28_N_4_O_4_: C 72.78; H 5.18; N 10.29; Found: C 72.63; H 5.01; N 10.44.

##### 2-(2-methoxy-4-((2-(2-phenylquinoline-4-carbonyl)hydrazineylidene)methyl)phenoxy)-N-(4-methoxyphenyl)acetamide (**11c**)

Yield: 84%. Brown solid. M.p. > 250 °C. IR (KBr): 3378, 3121, 1680, 1549, 1342, 1170, 989 cm^−1^. TLC (40:60; Hexane/AcOEt); Rf = 0.52**.** ^1^H NMR (400 M*Hz*, DMSO-*d*_*6*_) δ 12.18 (s, 1H, –NH–N=), 10.02 (s, 1H,NH), 8.50–8.37 (m, 1H, H_Ar_), 8.36 (s, 1H, H_Ar_), 8.32 (d, *J* = 7.8 *Hz*, 1H, H_Ar_), 8.25 (d, *J* = 8.2 *Hz*, 1H, H_Ar_), 8.19 (d, *J* = 8.3 *Hz*, 1H, H_Ar_), 7.87 (d, *J* = 8.4 *Hz*, 1H, H_Ar_), 7.69 (d, *J* = 8.6 *Hz*, 1H, H_Ar_), 7.60 (d, *J* = 8.6 *Hz*, 2H, H_Ar_), 7.58–7.54 (m, 3H, H_Ar_), 7.49–7.46 (m, 1H, H_Ar_), 7.26 (d, *J* = 8.3, 1H, H_Ar_), 7.05 (d, *J* = 8.4 *Hz*, 1H, H_Ar_), 6.92 (d, *J* = 8.9 *Hz*, 1H, H_Ar_), 4.76 (s, 2H, OCH_2_), 3.92 (s, 3H, OCH_3_), 3.73 (s, 3H, OCH_3_). ^13^C NMR (101 M*Hz*, DMSO-*d*_*6*_) δ 166.25, 163.13, 156.25, 155.98, 150.12, 149.87, 149.21, 148.40, 141.92, 138.55, 131.97, 130.86, 130.48, 130.13, 129.42, 127.81, 125.62, 122.47, 121.51, 114.39, 113.96, 109.26, 68.53, 56.10, 55.64. SI–MS (C_33_H_28_N_4_O_5_): calculated m/z 560.21 [M+H]^+^, observed m/z 560.23 [M+H]^+^; Anal. Calcd for C_33_H_28_N_4_O_5_: C, 70.70; H, 5.03; N, 9.99; Found: C 71.13; H 5.17; N 10.17.

##### N-(4-ethylphenyl)-2-(2-methoxy-4-((2-(2-phenylquinoline-4-carbonyl)hydrazono)methyl)phenoxy)acetamide (**11d**)

Yield: 76%. Brown solid. M.p. > 250 °C. IR (KBr): 3419, 3074, 1659, 1623, 1357, 1169 cm^−1^. TLC (40:60; Hexane/AcOEt); Rf = 0.48.^1^H NMR (400 M*Hz*, DMSO-*d*_*6*_) δ 12.17 (s, 1H, –NH–N=), 10.08 (s, 1H,NH), 8.49–8.33 (m, 3H, H_Ar_), 8.31 (s, 1H, H_Ar_), 8.24 (d, *J* = 8.3 *Hz*, 1H, H_Ar_), 8.18 (d, *J* = 8.2 *Hz*, 1H, H_Ar_), 7.87 (d, *J* = 8.4 *Hz*, 1H, H_Ar_), 7.69 (d, *J* = 8.3 *Hz*, 1H, H_Ar_), 7.61–7.56 (m, 2H, H_Ar_), 7.54 (d, *J* = 8.7 *Hz*, 2H, H_Ar_), 7.51–7.45 (m, 1H, H_Ar_), 7.25 (d, *J* = 9.1 *Hz*, 1H, H_Ar_), 7.17 (d, *J* = 8.4 *Hz*, 2H, H_Ar_), 7.04 (d, *J* = 8.3 *Hz*, 1H, H_Ar_), 4.77 (s, 2H, OCH_2_), 3.92 (s, 3H, OCH_3_), 2.69–2.53 (m, 2H,CH_2Ethyl_), 1.16 (t, *J* = 7.6 *Hz*, 3H, CH_3Ethyl_). ^13^C NMR (101 M*Hz*, DMSO-*d*_*6*_) δ 166.50, 163.11, 156.25, 150.09, 149.85, 148.39, 139.58, 138.54, 136.56, 130.87, 130.49, 129.43, 125.62, 123.96, 122.45, 119.99, 117.70, 113.92, 109.26, 68.46, 56.10, 28.08, 16.19. SI–MS (C_34_H_30_N_4_O_4_): calculated m/z 560.21 [M+H]^+^, observed m/z 560.29 [M+H]^+^; Anal. Calcd for C_34_H_30_N_4_O_4_: C, 73.10; H, 5.41; N, 10.03; O, 11.46; Found: C 73.54; H 5.47; N 10.09.

##### N-(4-fluorophenyl)-2-(2-methoxy-4-((2-(2-phenylquinoline-4-carbonyl)hydrazono)methyl)phenoxy)acetamide (**11e**)

Yield: 79%. Brown solid. M.p. > 250 °C. IR (KBr): 3431, 3012, 1688, 1552, 1332, 1178, 925 cm^−1^. TLC (40:60; Hexane/AcOEt); Rf = 0.38.^1^H NMR (400 M*Hz*, DMSO-*d*_*6*_ ) δ 12.19 (s, 1H, –NH–N=), 10.24 (s, 1H,NH), 8.38–8.24 (m, 4H, H_Ar_), 8.22 (d, *J* = 8.4*Hz*, 1H, H_Ar_), 8.19 (d, *J* = 8.3 *Hz*, 1H, H_Ar_), 7.89–7.85 (m, 1H, H_Ar_), 7.70–7.66 (m, 2H, H_Ar_), 7.58–7.48 (m, 3H, H_Ar_), 7.49 (d, *J* = 8.1 *Hz*, 1H, H_Ar_), 7.26 (d, *J* = 8.4, 1H, H_Ar_), 7.19 (t, *J* = 8.9 *Hz*, 2H, H_Ar_), 7.05 (d, *J* = 8.3 *Hz*, 1H, H_Ar_), 4.80 (s, 2H, OCH_2_), 3.91 (s, 3H, OCH_3_).^13^C NMR (100 M*Hz*, DMSO-*d*_*6*_) δ 166.72, 163.15, 159.89 (^1^*J*_C-F_ = 239 *Hz*), 156.24, 150.05, 149.87, 149.21, 148.39, 141.89, 138.54, 135.27 (^4^*J*_C-F_ = 2 *Hz*), 130.84, 130.47, 130.13, 129.41, 128.18, 127.91, 127.80, 125.61, 123.96, 122.46, 121.81 (^3^*J*_C-F_ = 8 *Hz*), 117.70, 115.97 (^2^*J*_C-F_ = 22 *Hz*), 113.97, 109.27, 68.45, 56.08. SI–MS (C_32_H_25_FN_4_O_4_): calculated m/z 548.57 [M+H]^+^, observed m/z 548.60 [M+H]^+^; *Anal*. Calcd for C_32_H_25_FN_4_O_4_: C 70.06; H 4.59; N 10.21; Found: C 69.89; H 4.47; N 10.02.

##### N-(4-chlorophenyl)-2-(2-methoxy-4-((2-(2-phenylquinoline-4-carbonyl)hydrazono)methyl)phenoxy)acetamide (**11f**)

Yield: 71%. Brown solid. M.p. > 250 °C. IR (KBr): 3443, 3031, 1684, 1587, 1324, 1206, 736 cm^−1^. TLC (40:60; Hexane/AcOEt); R_f_ = 0.48.^1^H NMR (400 M*Hz*, DMSO-*d*_*6*_) δ 12.27 (s, 1H, –NH–N=), 10.42 (s, 1H,NH), 8.43–8.29 (m, 4H, H_Ar_), 8.21 (d, *J* = 8.5 *Hz*, 2H, H_Ar_), 7.87 (t, *J* = 7.8 *Hz*, 1H, H_Ar_), 7.70–7.55 (m, 6H, H_Ar_), 7.47 (t,* J* = 7.9 *Hz*, 1H, H_Ar_), 7.40 (d, *J* = 8.5 *Hz*, 2H, H_Ar_), 7.25 (d, *J* = 8.2 *Hz*, 1H, H_Ar_), 7.04 (d, *J* = 8.3 *Hz*, 1H, H_Ar_), 4.81 (s, 2H, OCH_2_), 3.91 (s, 3H, OCH_3_).^13^C NMR (101 M*Hz*, DMSO-*d*_*6*_) δ 166.97, 163.15, 156.24, 150.02, 149.84, 149.21, 148.38, 141.90, 138.55, 137.89, 130.83, 130.46, 130.12, 129.41, 129.18, 128.18, 127.90, 127.80, 127.69, 125.62, 123.96, 122.44, 121.49, 117.71, 113.92, 109.26, 68.38, 56.09. SI–MS (C_32_H_25_ClN_4_O_4_): calculated m/z 5654.03 [M+H]^+^, observed m/z 565.05 [M+H]^+^; *Anal*. Calcd for C_32_H_25_ClN_4_O_4_: C 68.02; H 4.46; N 9.29; Found: C 67.88; H 4.23; N 9.11.

##### N-(4-bromophenyl)-2-(2-methoxy-4-((2-(2-phenylquinoline-4-carbonyl)hydrazineylidene)methyl)phenoxy)acetamide (**11g**)

Yield: 74%. Brown solid. M.p. > 250 °C. IR (KBr): 3451, 3018, 1677, 1563, 1320, 1192, 668 cm^−1^. TLC (40:60; Hexane/AcOEt); Rf = 0.39.^1^H NMR (400 M*Hz*, DMSO-*d*_*6*_) δ 12.25 (s, 1H, –NH–N=), 10.39 (s, 1H,NH), 8.38–8.18 (m, 6H, H_Ar_), 7.86 (t, *J* = 7.9 *Hz*, 1H, H_Ar_), 7.70–7.48 (m, 9H, H_Ar_), 7.25 (d, *J* = 8.2 *Hz*, 1H, H_Ar_), 7.05 (d, *J* = 8.2 *Hz*, 1H, H_Ar_), 4.82 (s, 2H, OCH_2_), 3.91 (s, 3H, OCH_3_).^13^C NMR (101 M*Hz*, DMSO-*d*_*6*_) δ 167.00, 163.17, 156.23, 150.03, 149.84, 149.23, 148.40, 141.87, 138.55, 138.30, 132.09, 130.82, 130.46, 130.13, 129.40, 128.18, 127.89, 127.80, 125.63, 123.97, 122.46, 121.87, 117.72, 115.76, 113.93, 109.25, 68.41, 56.08. SI–MS (C_32_H_25_BrN_4_O_4_): calculated m/z 609.48 [M+H]^+^, observed m/z 609.51 [M+H]^+^; *Anal*. Calcd for C_32_H_25_BrN_4_O_4_: C 63.06; H 4.13; N 9.19; Found: C 62.85; H 4.31; N 9.39.

##### 2-(2-methoxy-4-((2-(2-(4-methoxyphenyl)quinoline-4-carbonyl)hydrazono)methyl)phenoxy)-N-phenylacetamide (**11h**)

Yield: 71%. Brown solid. M.p. > 250 °C. IR (KBr): 3453, 3027, 1660, 1585, 1344, 1229 cm^−1^. TLC (40:60; Hexane/AcOEt); R_f_ = 0.50.^1^H NMR (400 M*Hz*, DMSO-*d*_*6*_) δ 12.27 (s, 1H, –NH–N=), 10.24 (s, 1H,NH), 8.36–8.30 (m,4H, H_Ar_), 8.21 (d, *J* = 8.4, 1H, H_Ar_), 8.14 (d, *J* = 8.4 *Hz*, 1H, H_Ar_), 7.83 (t, *J* = 7.6, 1H, H_Ar_), 7.66 (d, *J* = 7.4 *Hz*, 2H, H_Ar_), 7.49 (d, *J* = 8.8 *Hz*, 1H, H_Ar_), 7.40–7.29 (m, 2H, H_Ar_), 7.25 (d, *J* = 8.3*Hz*, 1H, H_Ar_), 7.15–7.04 (m, 5H, H_Ar_), 4.82 (s, 2H, OCH_2_), 3.89 (m, 6H, 2XOCH_3_).^13^C NMR (101 M*Hz*, DMSO-*d*_*6*_) δ 166.76, 163.31, 161.38, 155.89, 150.06, 149.84, 149.19, 148.39, 141.78, 138.91, 130.98, 130.69, 129.89, 129.29, 129.22, 128.17, 127.39, 125.59, 124.13, 123.63, 122.46, 119.90, 117.21, 114.76, 113.88, 109.22, 68.43, 56.08, 55.80. SI–MS (C_33_H_28_N_4_O_5_): calculated m/z 560.61 [M+H]^+^, observed m/z 560.64 [M+H]^+^; *Anal*. Calcd for C_33_H_28_N_4_O_5_: C 70.70; H 5.03; N 9.99; Found: C 70.52; H 5.21; N 10.18.

##### 2-(2-methoxy-4-((2-(2-(4-methoxyphenyl)quinoline-4-carbonyl)hydrazono)methyl)phenoxy)-N-(o-tolyl)acetamide (**11i**)

Yield: 71%. Brown solid. M.p. > 250 °C. IR (KBr): 3439, 3011, 1648, 1575, 1328, 1164 cm^−1^. TLC (40:60; Hexane/AcOEt); Rf = 0.45.^1^H NMR (400 M*Hz*, DMSO-*d*_*6*_) δ 12.27 (s, 1H, –NH–N=), 9.43 (s, 1H,NH), 8.36–8.06 (m, 6H, H_Ar_), 7.83 (t, *J* = 7.9 *Hz*, 1H, H_Ar_), 7.71–7.38 (m, 3H, H_Ar_), 7.34–7.01 (m, 7H, H_Ar_), 4.84 (s, 2H, OCH_2_), 3.92 (s, 3H, OCH_3_), 3.87 (s, 3H, OCH_3_), 2.24 (s, 3H,CH_3_).^13^C NMR (101 M*Hz*, DMSO-*d*_*6*_) δ 166.71, 163.29, 161.39, 155.89, 149.82, 149.73, 149.12, 148.38, 141.81, 136.02, 131.25, 130.98, 130.85, 130.79, 130.70, 129.29, 128.30, 127.40, 126.62, 125.69, 125.59, 124.38, 123.63, 122.34, 117.20, 114.77, 113.92, 109.28, 68.24, 56.14, 55.82, 17.96. SI–MS (C_34_H_30_N_4_O_5_): calculated m/z 574.64O [M+H]^+^, observed m/z 574.66 [M + H]^+^; *Anal*. Calcd for C_34_H_30_N_4_O_5_: C 71.07; H 5.26; N 9.75; Found: C 70.90; H 5.46; N 9.58.

##### 2-(2-methoxy-4-((2-(2-(4-methoxyphenyl)quinoline-4-carbonyl)hydrazono)methyl)phenoxy)-N-(4-methoxyphenyl)acetamide (**11j**)

Yield: 76%. Brown solide. M.p. > 250 °C. IR (KBr): 3458, 3025, 1638, 1581, 1326, 1212 cm^−1^. TLC (40:60; Hexane/AcOEt); Rf = 0.58.^1^H NMR (400 M*Hz*, DMSO-*d*_*6*_) δ 12.17 (s, 1H, –NH–N=), 10.02 (s, 1H,NH), 8.36–8.30 (m, 3H, H_Ar_), 8.18 (d, *J* = 8.2 *Hz*, 1H, 1H, H_Ar_), 8.13 (d, *J* = 8.4 *Hz*, 1H, H_Ar_), 7.83 (t,* J* = 8.00 *Hz*, 1H, H_Ar_), 7.64 (t, *J* = 7.5, 1H, H_Ar_), 7.55 (d, *J* = 8.9 *Hz*, 2H, H_Ar_), 7.47 (d, *J* = 8.2 *Hz*, 1H, H_Ar_), 7.25 (d, *J* = 8.4*Hz*, 1H, H_Ar_), 7.14 (d, *J* = 8.8 *Hz*, 2H, H_Ar_), 7.05 (d, *J* = 8.3 *Hz*, 1H, H_Ar_), 6.92 (d, *J* = 8.7 *Hz*, 2H, H_Ar_), 4.76 (s, 2H, OCH_2_), 3.91 (s, 3H, OCH_3_), 3.87 (s, 3H, OCH_3_), 3.73 (s, 3H, OCH_3_).^13^C NMR (101 M*Hz*, DMSO-*d*_*6*_) δ 166.24, 163.22, 161.39, 155.96, 155.88, 150.08, 149.85, 149.13, 148.37, 141.77, 131.96, 130.96, 130.72, 129.89, 129.29, 129.13, 128.14, 127.42, 125.56, 123.60, 122.43, 121.49, 117.18, 114.77, 114.38, 109.24, 68.50, 56.09, 55.82, 55.64. SI–MS (C_34_H_30_N_4_O_6_): calculated m/z 590.64 [M+H]^+^, observed m/z 590.61 [M+H]^+^; *Anal*. Calcd for C_34_H_30_N_4_O_6_: C 69.14; H 5.12; N 9.49; Found: C 68.96; H 4.91; N 9.67.

##### N-(4-ethylphenyl)-2-(2-methoxy-4-((2-(2-(4-methoxyphenyl)quinoline-4-carbonyl)hydrazono)methyl)phenoxy)acetamide (**11k**)

Yield: 68%. Brown solid. M.p. > 250 °C. IR (KBr): 3446, 3014, 1657, 1593, 1335, 1183 cm^−1^. TLC (40:60; Hexane/AcOEt); R_f_ = 0.51^. 1^H NMR (400 MHz, DMSO-*d*_*6*_) δ 12.18 (s, 1H, –NH–N=), 10.08 (s, 1H, NH), 8.36–8.30 (m, 4H, N = CH, H_3,_ H_2′_,_6′_), 8.20 (d, *J* = 8.2 Hz, 1H, H_5_), 8.14 (d, *J* = 8.4 Hz, 1H, H_8_), 7.83 (t, *J* = 7.8 Hz, 1H, H_7_), 7.64 (t, J = 7.6 Hz, 1H, H_6_), 7.56 (d, *J* = 8.3 Hz, 2H, H_2,6acetamide,_), 7.49 (s, 1H, H_2″_) 7.26 (d, *J* = 8.2 Hz, 1H, H_6″_), 7.18–7.13 (m, 4H, H_3,5acetamide_, H_3′_,_5′_), 7.05 (d, *J* = 8.3 Hz, 1H, H_5″_), 4.79 (s, 2H, CH_2acetamide_), 3.91 (s, 3H, OCH_3_), 3.86 (s, 3H, OCH_3_), 2.56 (q,* J* = 7.6 Hz, 2H, CH_2Ethyl_), 1.16 (t, *J* = 7.6 Hz, 3H, _CH3Ethyl_).^13^C NMR (101 MHz, DMSO-*d*_*6*_) δ 166.51, 163.25, 161.39, 155.89, 150.08, 149.85, 149.16, 148.39, 141.74, 139.59, 136.54, 130.97, 130.71, 129.29, 128.47, 128.15, 127.41, 125.56, 123.61, 122.45, 120.00, 119.96, 117.19, 114.77, 113.92, 109.24, 68.49, 56.08, 55.80, 28.09, 16.18. SI–MS (C_35_H_32_N_4_O_5_): calculated m/z 588.24 [M+H]^+^, observed m/z 588.45 [M+H]^+^; Anal. Calcd for C_35_H_32_N_4_O_5_: C 71.41; H 5.48; N 9.52; Found: C 71.21; H 5.66; N 9.34.

##### N-(4-fluorophenyl)-2-(2-methoxy-4-((2-(2-(4-methoxyphenyl)quinoline-4-carbonyl)hydrazono)methyl)phenoxy)acetamide (**11l**)

Yield: 83%. Brown solid. M.p. > 250 °C. IR (KBr): 3455, 3039, 1674, 1603, 1341, 1175, 991 cm^−1^. TLC (40:60; Hexane/AcOEt); Rf = 0.54.^1^H NMR (400 M*Hz*, DMSO-*d*_*6*_) δ 12.19 (s, 1H, –NH–N=), 10.25 (s, 1H,NH), 8.36–8.32 (m, 4H, H_Ar_), 8.20 (d, *J* = 8.7 *Hz*, 1H, H_Ar_), 8.14 (d, *J* = 8.4 *Hz*, 1H, H_Ar_), 7.84 (t,* J* = 8.1 *Hz*, 1H, H_Ar_), 7.70–7.62 (m, 3H, H_Ar_), 7.49 (d, *J* = 8.2 *Hz*, 1H, H_Ar_), 7.26 (d, *J* = 8.3, 1H, H_Ar_), 7.21–7.13 (m, 4H, H_Ar_), 7.05 (d, *J* = 8.3 *Hz*, 1H, H_Ar_), 4.80 (s, 2H, OCH_2_), 3.89 (m, 6H, 2xOCH_3_).^13^C NMR (101 M*Hz*, DMSO-*d*_*6*_) δ 166.73, 163.26, 161.39, 159.89 (^1^*J*_C-F_ = 239 *Hz*), 155.88, 150.03, 149.85, 149.15, 148.39, 141.74, 135.28 (^4^*J*_C-F_ = 3 *Hz*), 130.96, 130.71, 129.90, 129.29, 128.18, 127.42, 125.56, 123.61, 122.45, 121.80 (^3^*J*_C-F_ = 8 *Hz*), 117.19, 115.97 (^2^*J*_C-F_ = 22 *Hz*), 114.76, 113.94, 109.23, 68.43, 56.07, 55.80. SI–MS (C_33_H_27_FN_4_O_5_): calculated m/z 578.60 [M+H]^+^, observed m/z 578.63 [M+H]^+^; *Anal*. Calcd for C_33_H_27_FN_4_O_5_: C 68.50; H 4.70; N 9.68; Found: C 68.35; H 4.57; N 9.42.

##### N-(4-bromophenyl)-2-(2-methoxy-4-((2-(2-(4-methoxyphenyl)quinoline-4-carbonyl)hydrazono)methyl)phenoxy)acetamide (**11m**)

Yield: 79%. Brown solid. M.p. > 250 °C. IR (KBr): 3462, 3030, 1669, 1579, 1338, 1223, 652 cm^−1^. TLC (40:60; Hexane/AcOEt); Rf = 0.49.^1^H NMR (400 M*Hz*, DMSO-*d*_*6*_) δ 12.21 (s, 1H, –NH–N=), 10.36 (s, 1H,NH), 8.36–8.8.31 (m, 4H, H_Ar_), 8.20 (d, *J* = 8.1 *Hz*, 1H, H_Ar_), 8.13 (d, *J* = 8.3 *Hz*, 1H, H_Ar_), 7.82 (t, *J* = 8.00 *Hz*, 1H, H_Ar_), 7.66–7.62 (m, 3H, H_Ar_), 7.53 (d, *J* = 8.7 *Hz*, 2H, H_Ar_), 7.48 (d, *J* = 8.5 *Hz*, 1H, H_Ar_), 7.25 (d, *J* = 8.4, 1H, H_Ar_), 7.14 (d, *J* = 8.7 *Hz*, 2H, H_Ar_), 7.04 (d, *J* = 8.3 *Hz*, 1H, H_Ar_), 4.81 (s, 2H, OCH_2_), 3.91 (s, 3H, OCH_3_), 3.86 (s, 3H, OCH_3_).^13^C NMR (101 M*Hz*, DMSO-*d*_*6*_) δ 166.99, 163.25, 161.39, 155.88, 150.00, 149.84, 149.15, 148.39, 141.74, 138.28, 132.10, 130.96, 130.70, 129.89, 129.29, 128.20, 127.41, 125.57, 123.61, 122.43, 121.86, 117.19, 115.76, 114.77, 113.94, 109.26, 68.42, 56.08, 55.81. SI–MS (C_33_H_28_N_4_O_4_): calculated m/z 639.51 [M+H]^+^, observed m/z 639.55 [M+H]^+^; *Anal*. Calcd for C_33_H_27_BrN_4_O_5_: C 61.98; H 4.26; N 8.76; Found: C 61.77; H 4.38; N 8.59.

##### N-(4-chlorophenyl)-2-(2-methoxy-4-((2-(2-(4-methoxyphenyl)quinoline-4-carbonyl)hydrazono)methyl)phenoxy)acetamide (**11n**)

Yield: 81%. Brown solid. M.p. > 250 °C. IR (KBr): 3449, 3022, 1675, 1594, 1346, 1219 cm^−1^. TLC (40:60; Hexane/AcOEt); R_f_ = 0.54.^1^H NMR (400 M*Hz*, DMSO-*d*_*6*_) δ 12.19 (s, 1H, –NH–N=), 10.33 (s, 1H,NH), 8.36–8.31 (m, 4H, H_Ar_), 8.20 (d, *J* = 8.2 *Hz*, 1H, H_Ar_), 8.14 (d, *J* = 8.3 *Hz*, 1H, H_Ar_), 7.83 (t, d, *J* = 8.1 *Hz*, 1H, H_Ar_), 7.70–7.61 (m, 3H, H_Ar_), 7.48 (d, *J* = 8.2 *Hz*, 1H, H_Ar_), 7.40 (d, *J* = 8.6 *Hz*, 2H, H_Ar_), 7.25 (d, *J* = 8.3, 1H, H_Ar_), 7.14 (d, *J* = 8.8 *Hz*, 2H, H_Ar_), 7.05 (d, *J* = 8.3 *Hz*, 1H, H_Ar_), 4.81 (s, 2H, OCH_2_), 3.91–3.86 (m, 6H, 2XOCH_3_).^13^C NMR (101 M*Hz*, DMSO-*d*_*6*_) δ 166.97, 163.25, 161.39, 155.88, 150.01, 149.85, 149.14, 148.39, 141.75, 137.85, 130.96, 130.71, 129.90, 129.29, 129.19, 128.20, 127.73, 127.41, 125.56, 123.61, 122.43, 121.49, 117.19, 114.77, 113.95, 109.27, 68.42, 56.08, 55.80. SI–MS (C_33_H_27_ClN_4_O_5_): calculated m/z 595.05 [M+H]^+^, observed m/z 595.07 [M+H]^+^; *Anal*. Calcd for C_33_H_27_ClN_4_O_5_: C 66.61; H 4.57; N 9.42; Found: C 66.33; H 4.29; N 9.24.

##### 2-(2-methoxy-4-((2-(2-(4-methoxyphenyl)quinoline-4-carbonyl)hydrazono)methyl)phenoxy)-N-(4-nitrophenyl)acetamide (**11o**)

Yield: 77%. Yellow solid. M.p. > 250 °C. IR (KBr): 3456, 3016, 1691, 1552, 1351, 1234 cm^−1^. TLC (40:60; Hexane/AcOEt); R_f_ = 0.32.^1^H NMR (400 M*Hz*, DMSO-*d*_*6*_) δ 12.24 (s, 1H, –NH–N=), 9.59 (s, 1H, NH), 8.36–8.30 (m, 4H, H_Ar_), 8.22–8.12 (m, 4H, H_Ar_), 8.13 (d, *J* = 8.4 *Hz*, 1H, H_Ar_), 7.85 (t, *J* = 7.8 *Hz*, 1H, H_Ar_), 7.65 (t, *J* = 7.6 *Hz*, 1H, H_Ar_), 7.51 (s, 1H, H_Ar_), 7.31 (d, *J* = 8.3 *Hz*, 1H, H_Ar_), 7.24 (d, *J* = 8.9 *Hz*, 2H, H_Ar_), 7.17–7.13 (m, 3H, H_Ar_), 5.05 (s, 2H, OCH_2_), 3.99 (s, 3H, OCH_3_), 3.87 (s, 3H, OCH_3_).^13^C NMR (101 M*Hz*, DMSO-*d*_*6*_) δ 166.31, 163.39, 161.40, 155.88, 149.72, 148.87, 148.37, 148.02, 145.08, 143.12, 141.82, 138.88, 130.94, 130.74, 129.90, 129.29, 128.29, 127.45, 126.37, 125.52, 123.57, 121.83, 117.19, 115.80, 114.78, 108.43, 67.79, 56.50, 55.82. SI–MS (C_33_H_27_N_5_O_7_): calculated m/z 605.60 [M+H]^+^, observed m/z 605.63 [M+H]^+^; *Anal*. Calcd for C_33_H_27_N_5_O_7_: C 65.45; H 4.49; N 11.56; Found: C 65.22; H 4.71; N 11.33.

### α-glucosidase inhibition assay

The anti-α-glucosidase effects of synthesized compounds were screened according to the previously reported method^22,23^.

### Enzyme kinetic studies

The mode of inhibition of the most potent derivative **11k**, identified with the lowest IC_50_, was investigated against an α-glucosidase activity at different concentrations of substrate, *p*-nitrophenyl *α*-d-glucopyranoside (1–10 mM), as in the absence and presence of **11k** at different concentrations (0, 6.5, 13, and 26 µM) as reported in our pressures^12,14,24^.

### Molecular dynamic (MD) simulation

In this study, the molecular dynamics (MD) simulation was carried out using the Desmond v5.3 module, which is integrated into the Maestro interface from Schrodinger 2018-4 suite^24^. The IFD method was used to obtain the appropriate pose for the MD simulation procedure of the compounds^25^. In order to conduct MD simulation, the first step involved solvating the protein–ligand complexes with explicit SPC water molecules and positioning them at the center of an appropriately sized orthorhombic box under Periodic Boundary Condition. To mimic real cellular ionic concentrations, counterions and a 0.15 M solution of NaCl were added to neutralize the system. The MD protocol consisted of three steps: minimization, pre-production, and production MD simulations. The system was allowed to relax for 2500 steps by the steepest descent approach to minimize the energy. Next, a small force constant was applied to the enzyme as the system's temperature was gradually raised from 0 to 300 K to prevent abrupt changes. MD simulations were executed under the NPT (constant number of atoms, constant pressure—1.01325 bar, and constant temperature—300 K) ensemble, utilizing the Nose–Hoover chain method as the default. Long-range electrostatic forces were calculated using the Particle-mesh-based Ewald approach, with a cutoff radius for Columbia forces set to 9.0 Å. The protein–ligand complex underwent 100 ns of production MD simulations, with data frames stored every 1000 ps during the simulation. The dynamic behavior and structural changes of the systems were analyzed by the calculation of the root mean square deviation (RMSD) and interaction diagram (Supplementary Information).

### Supplementary Information

Supplementary Information.

## Data Availability

All data generated or analysed during this study are included in this published article.
