# Supplementary material for: Aryl-quinoline-4-carbonyl hydrazone bearing different 2-methoxyphenoxyacetamides as potent α-glucosidase inhibitors; molecular dynamics, kinetic and structure–activity relationship studies
Source: Sci Rep. 2024 Jan 3;14:388. doi: 10.1038/s41598-023-50395-8 (PMC10764907; doi:10.1038/s41598-023-50395-8)

11a: 2-(2-methoxy-4-((2-(2-phenylquinoline-4-carbonyl)hydrazineylidene)methyl)phenoxy)-N-(o-tolyl)acetamide:

^
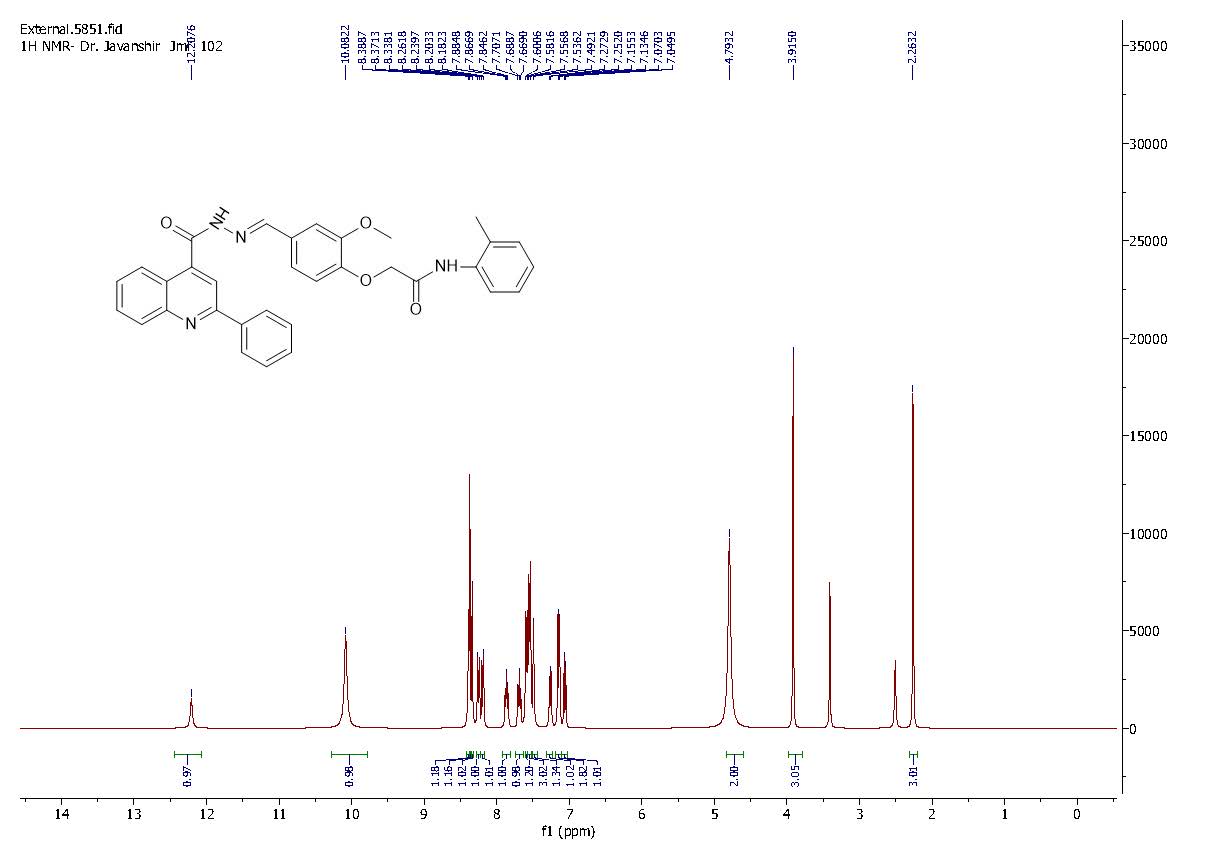
^


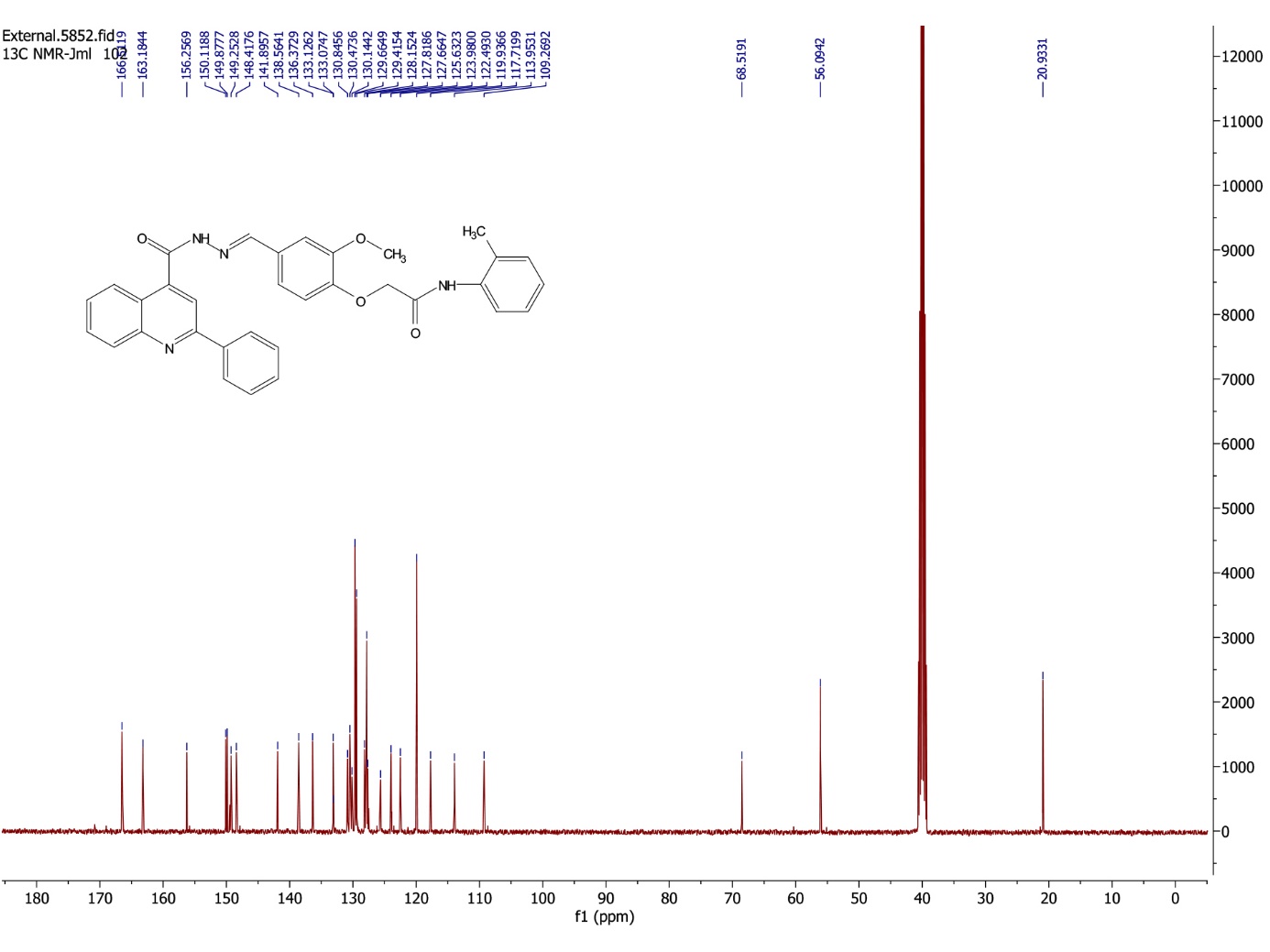


11b: 2-(2-methoxy-4-((2-(2-phenylquinoline-4-carbonyl)hydrazineylidene)methyl)phenoxy)-N-(p-tolyl)acetamide:


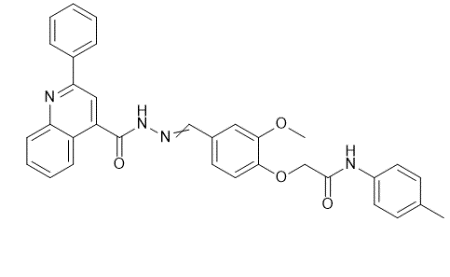

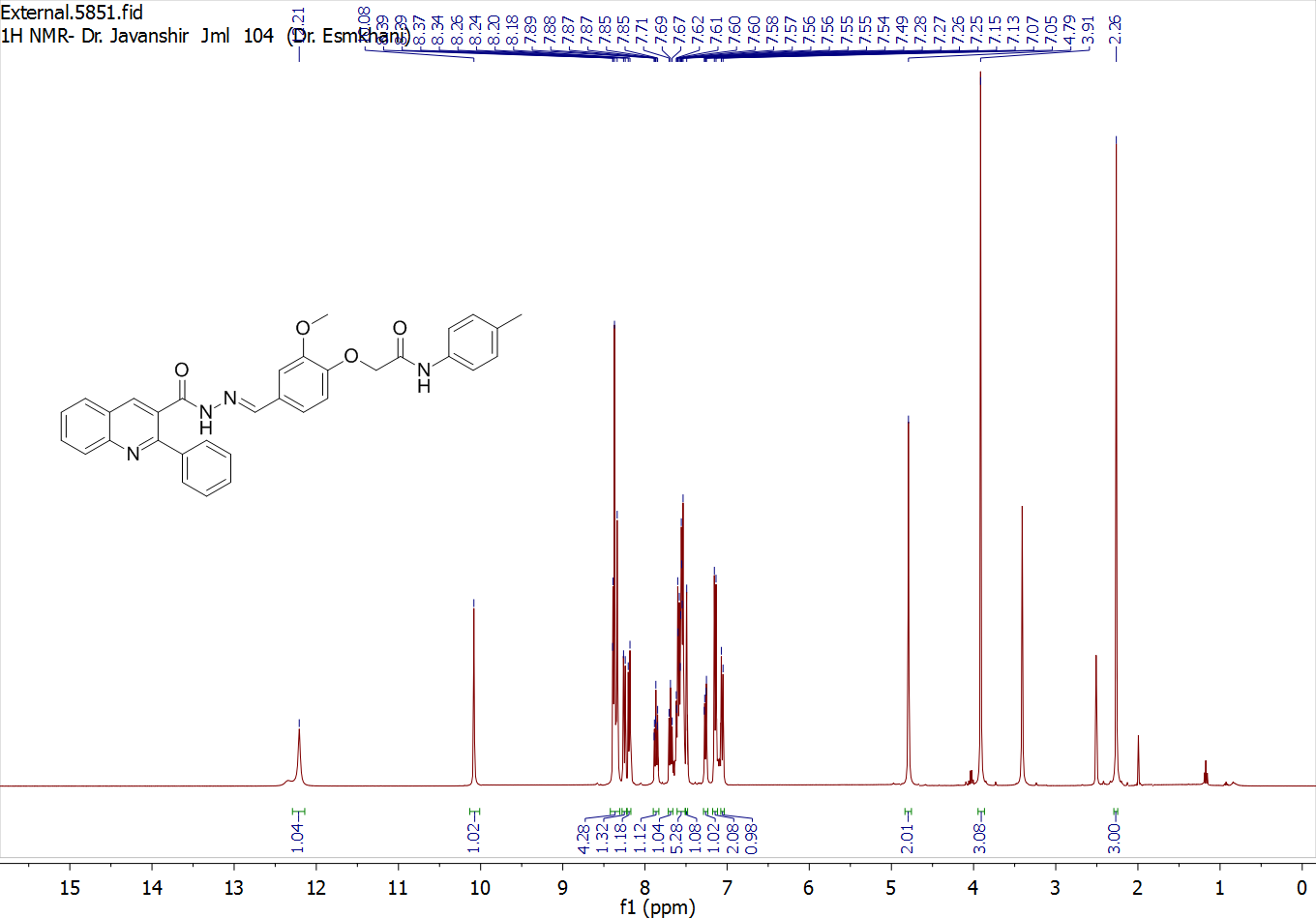


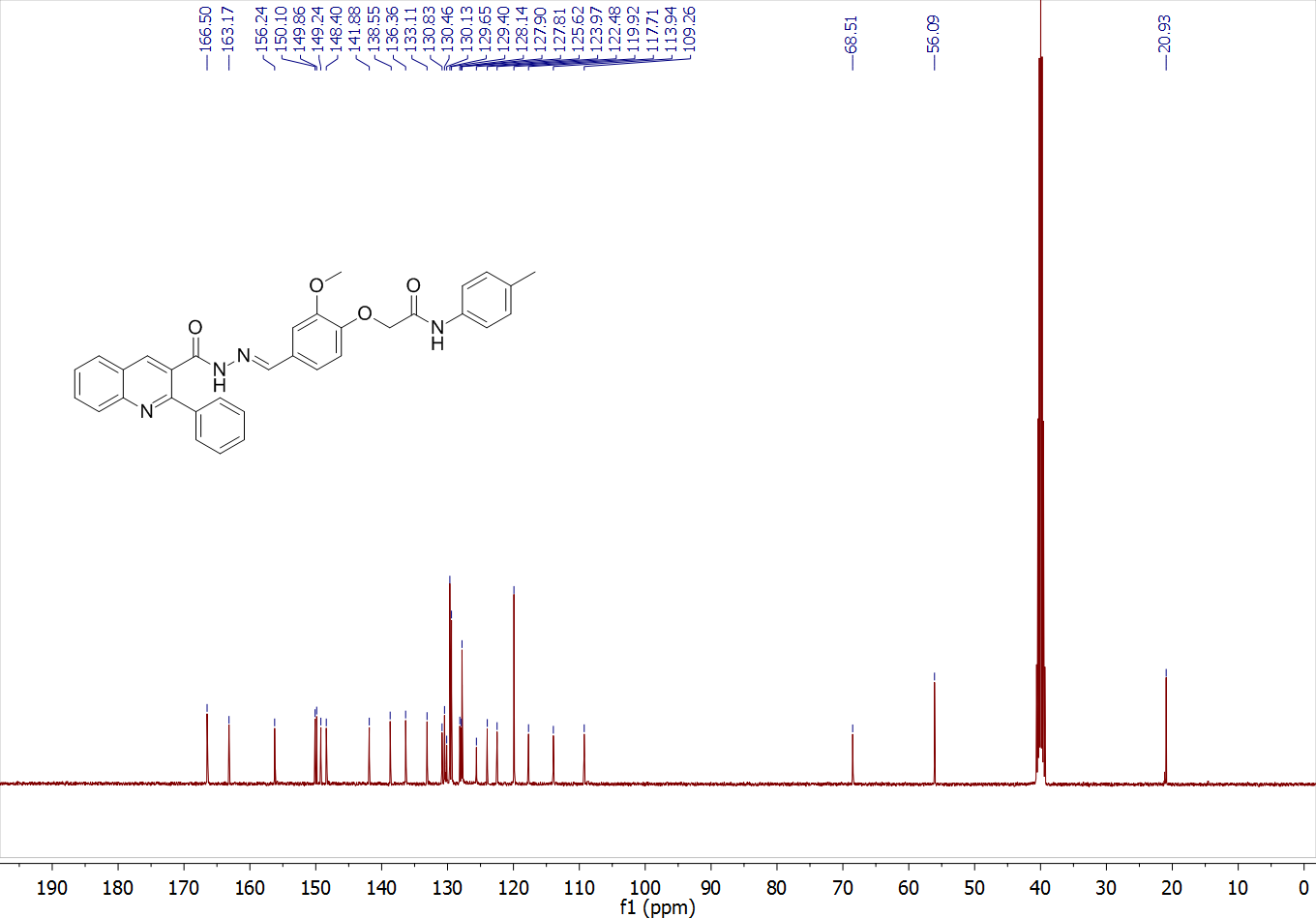


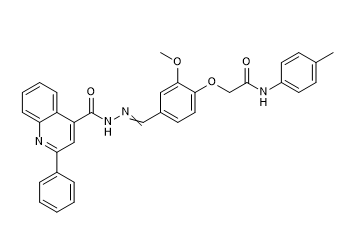


11c: 2-(2-methoxy-4-((2-(2-phenylquinoline-4-carbonyl)hydrazineylidene)methyl)phenoxy)-N-(4-methoxyphenyl)acetamide:


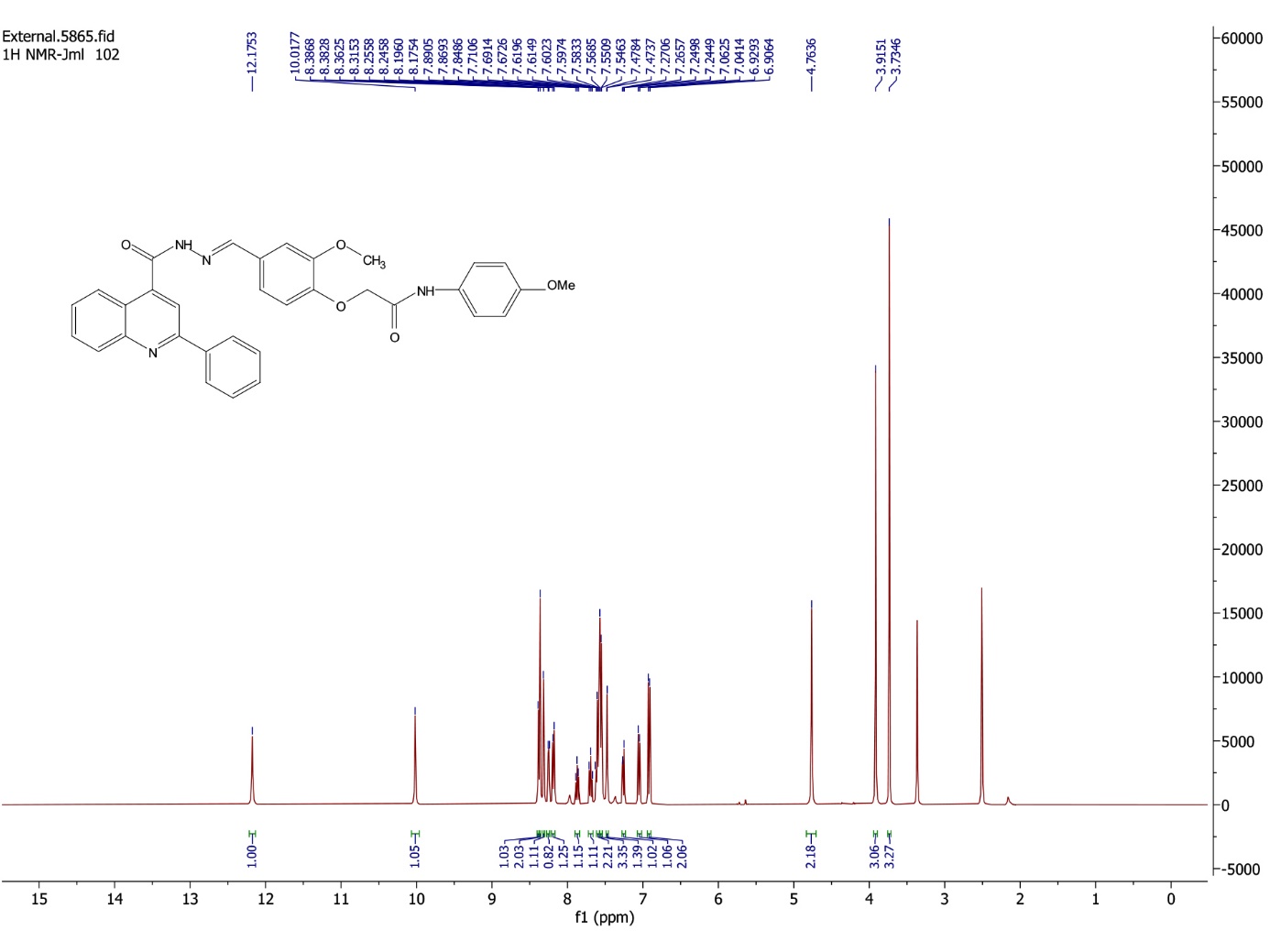


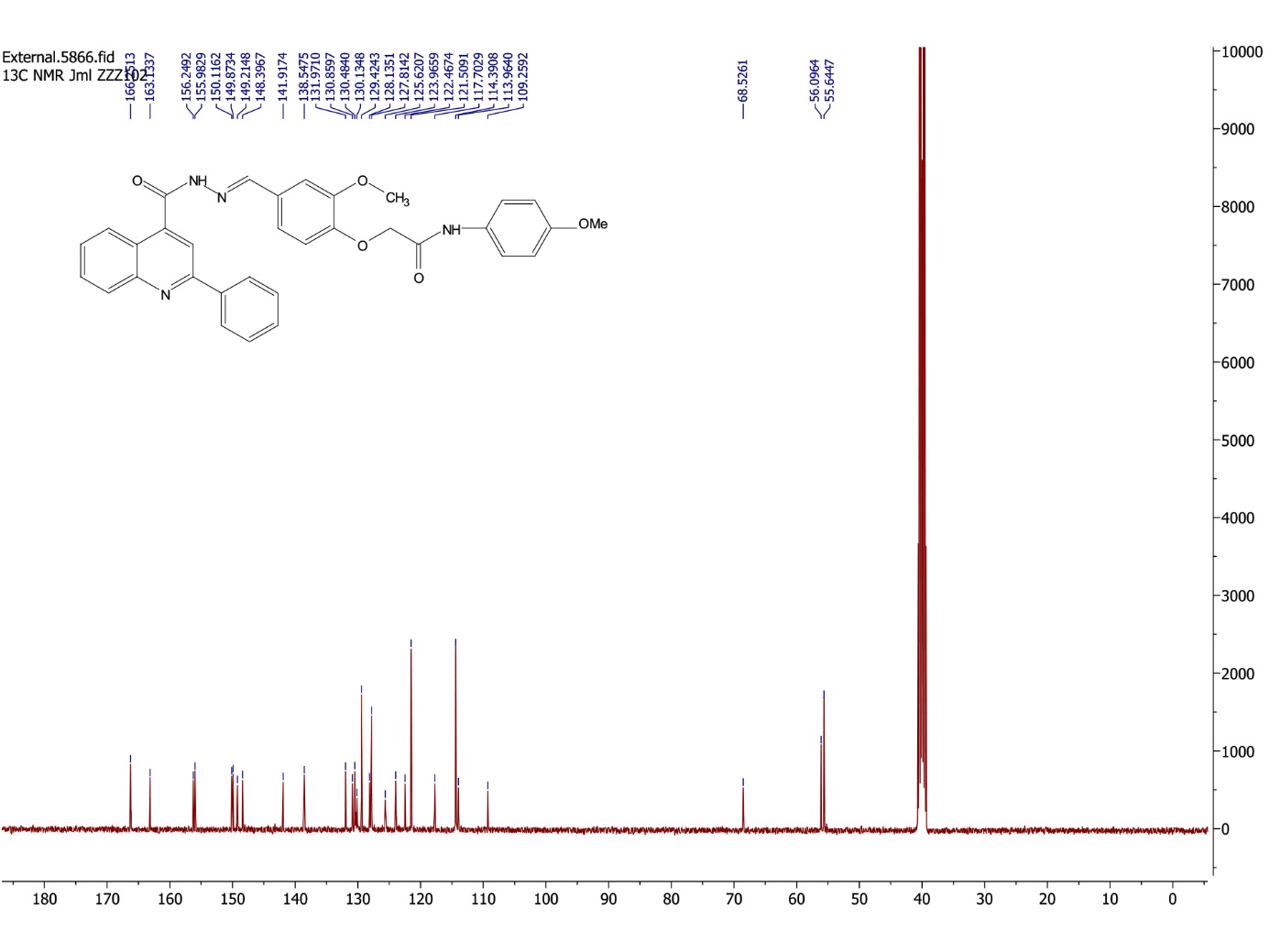


11d: N-(4-ethylphenyl)-2-(2-methoxy-4-((2-(2-phenylquinoline-4-carbonyl)hydrazineylidene)methyl)phenoxy)acetamide:


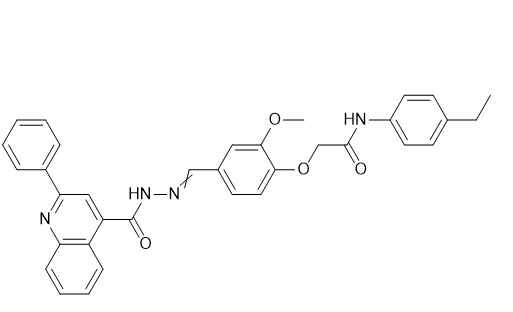

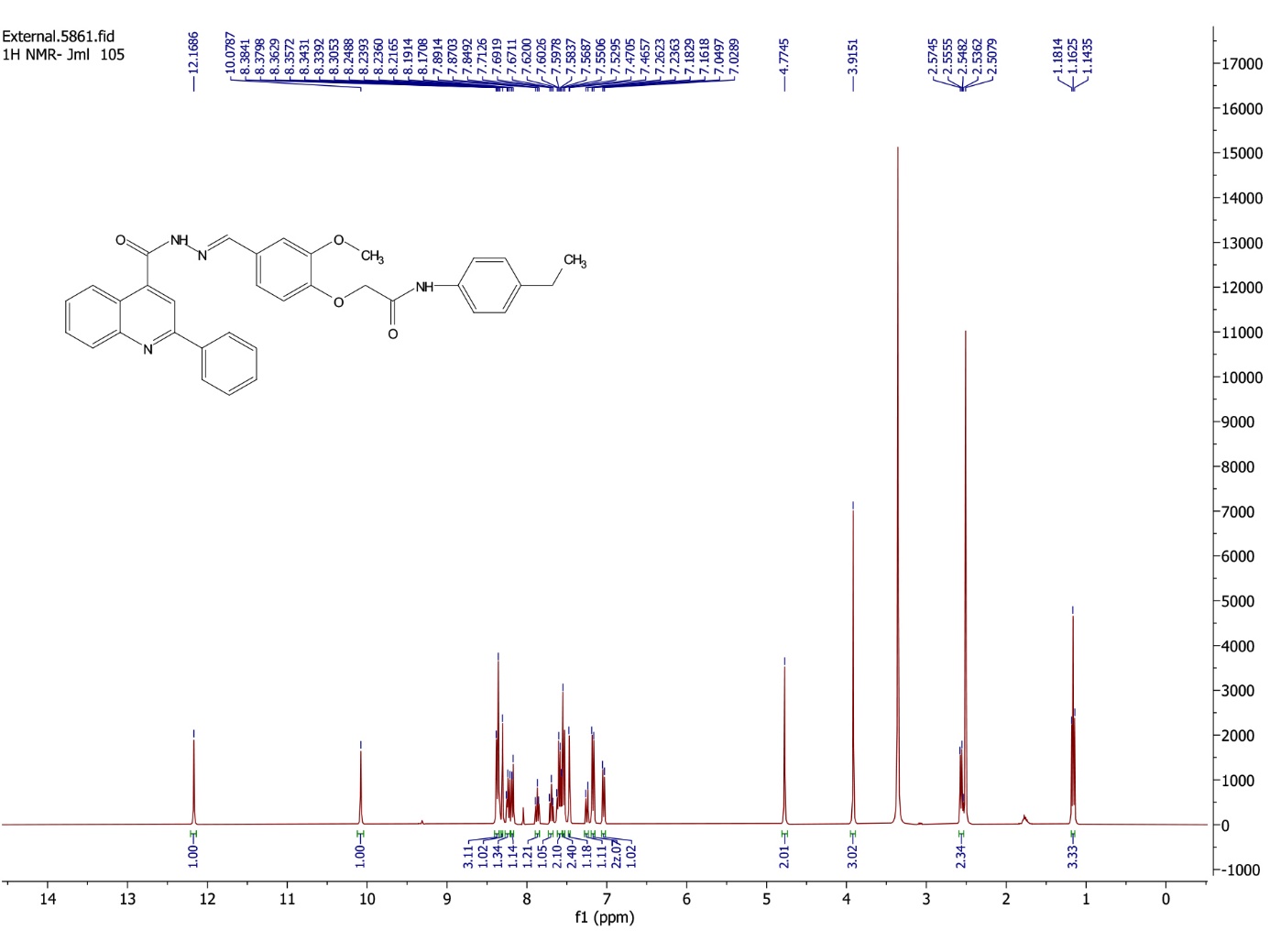


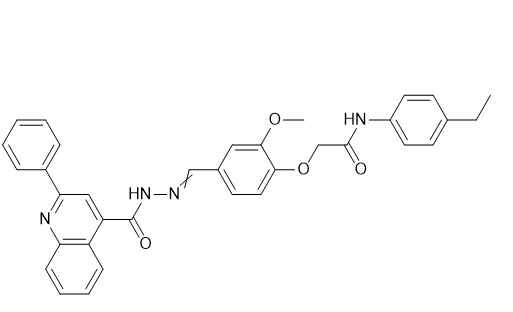

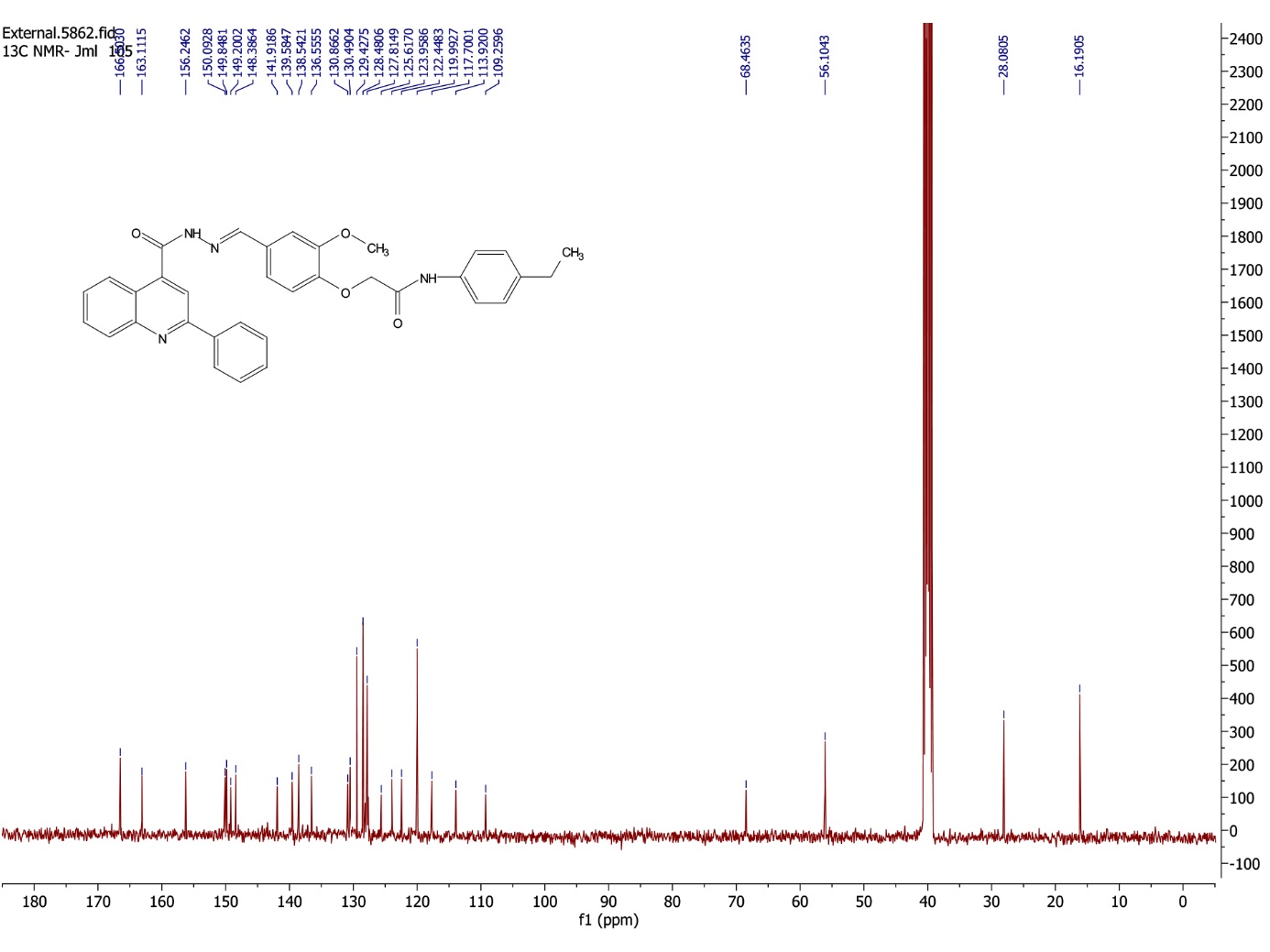


11e: N-(4-fluorophenyl)-2-(2-methoxy-4-((2-(2-phenylquinoline-4-carbonyl)hydrazineylidene)methyl)phenoxy)acetamide:


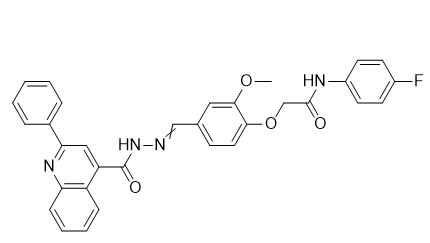

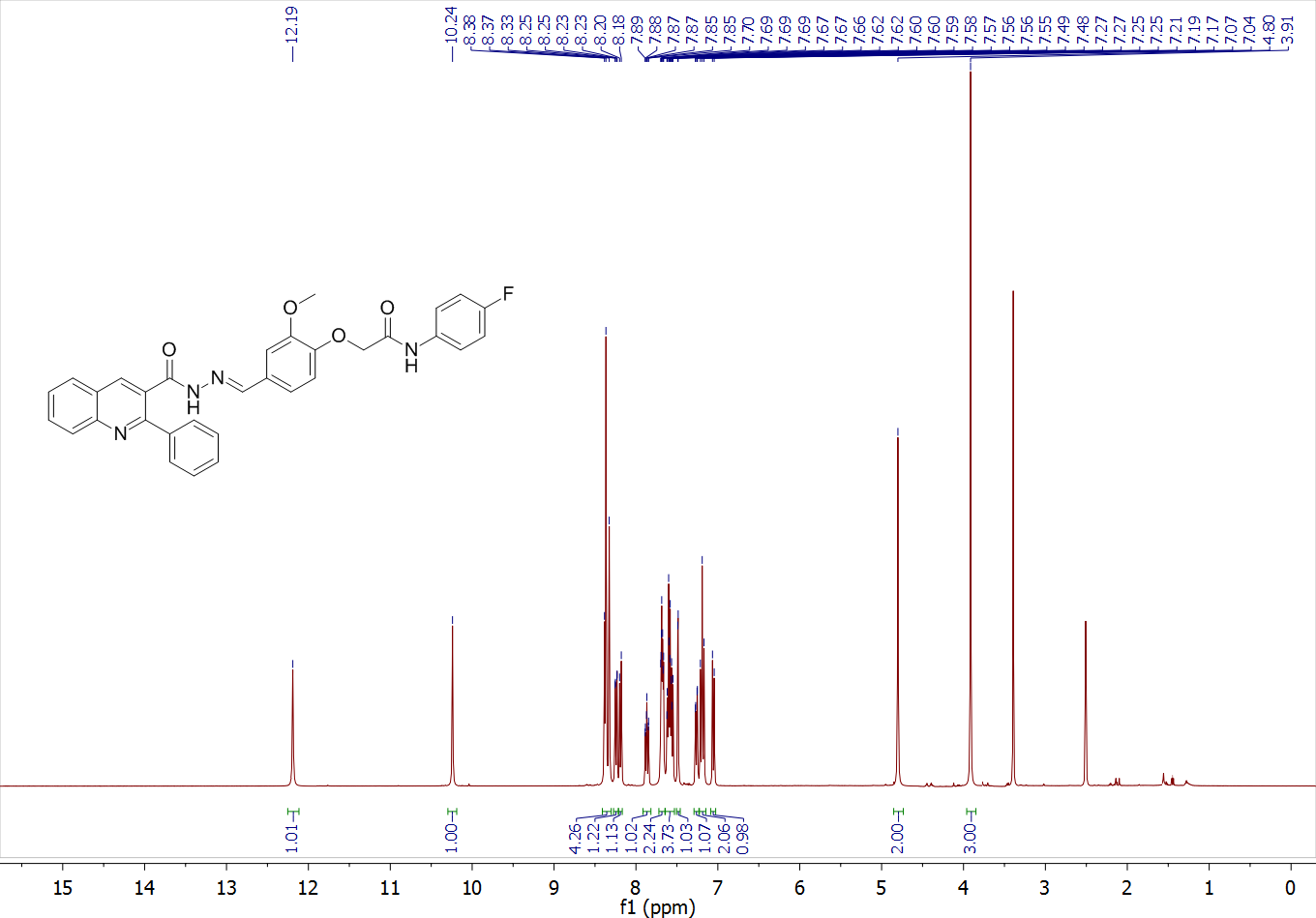


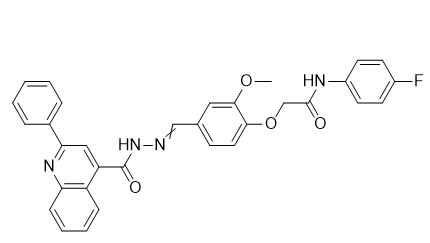

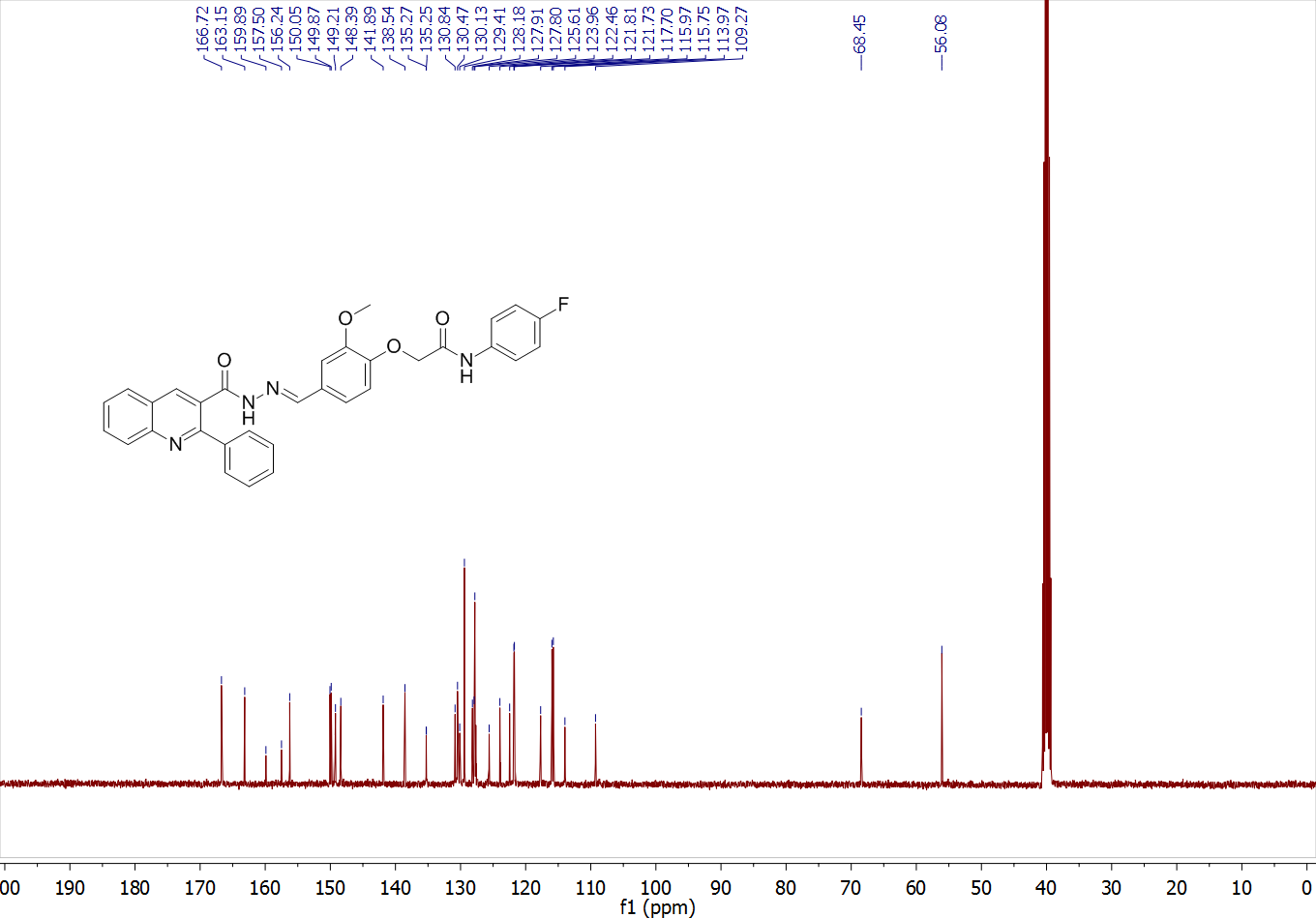


11f: N-(4-chlorophenyl)-2-(2-methoxy-4-((2-(2-phenylquinoline-4-carbonyl)hydrazineylidene)methyl)phenoxy)acetamide:


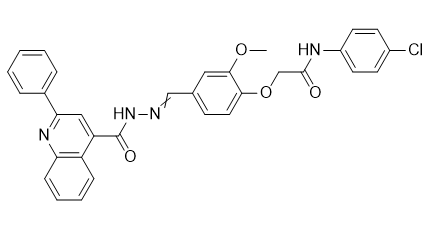

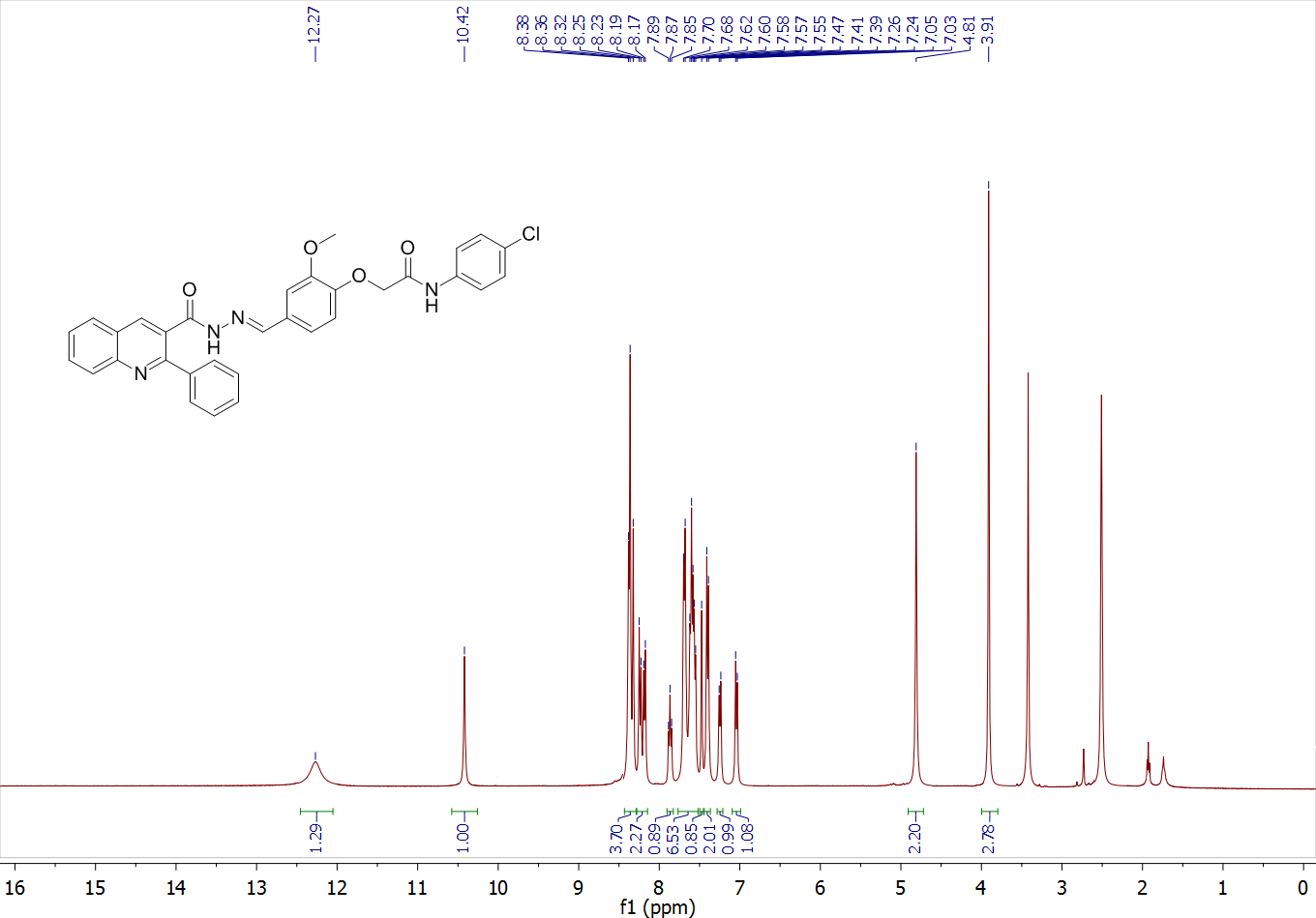


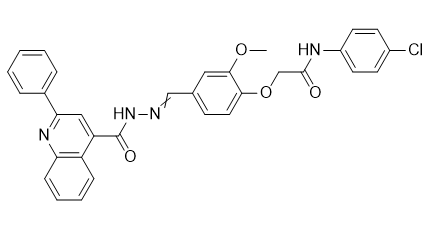

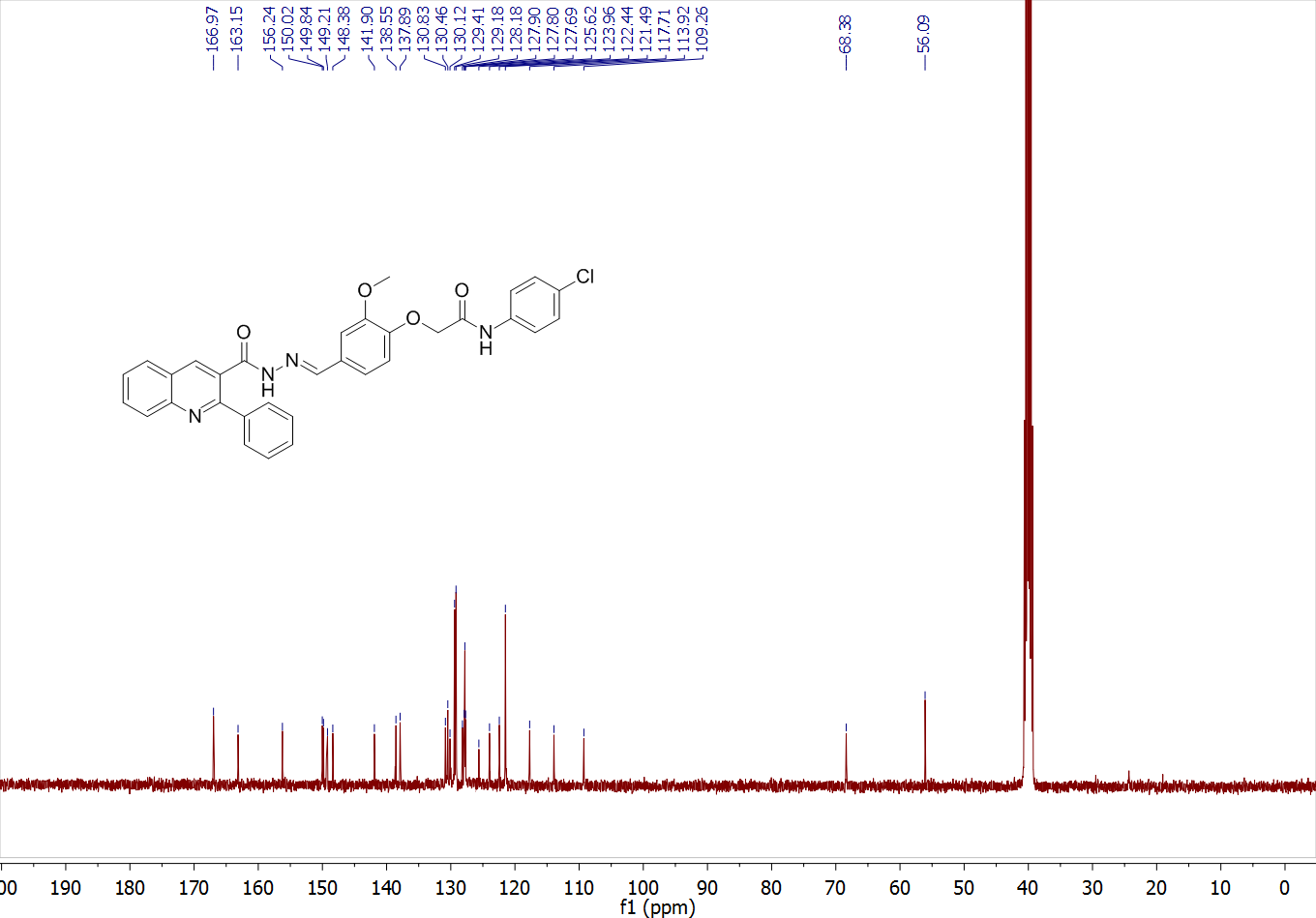


11g: N-(4-bromophenyl)-2-(2-methoxy-4-((2-(2-phenylquinoline-4-carbonyl)hydrazineylidene)methyl)phenoxy)acetamide:


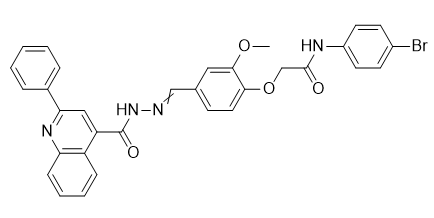

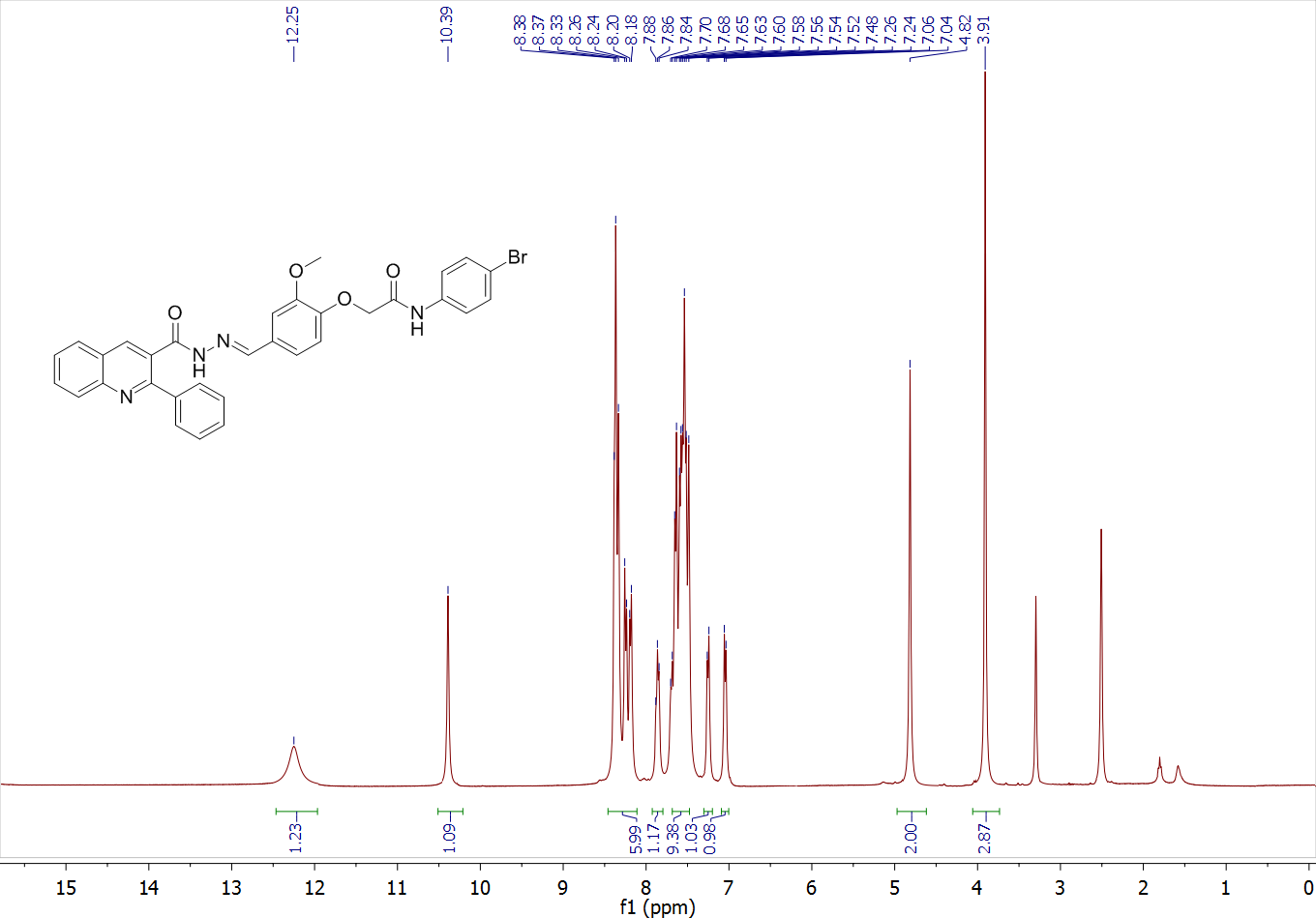


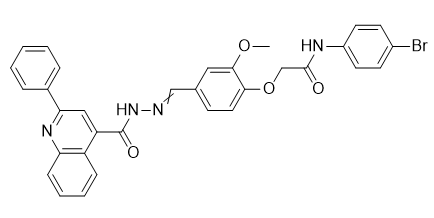

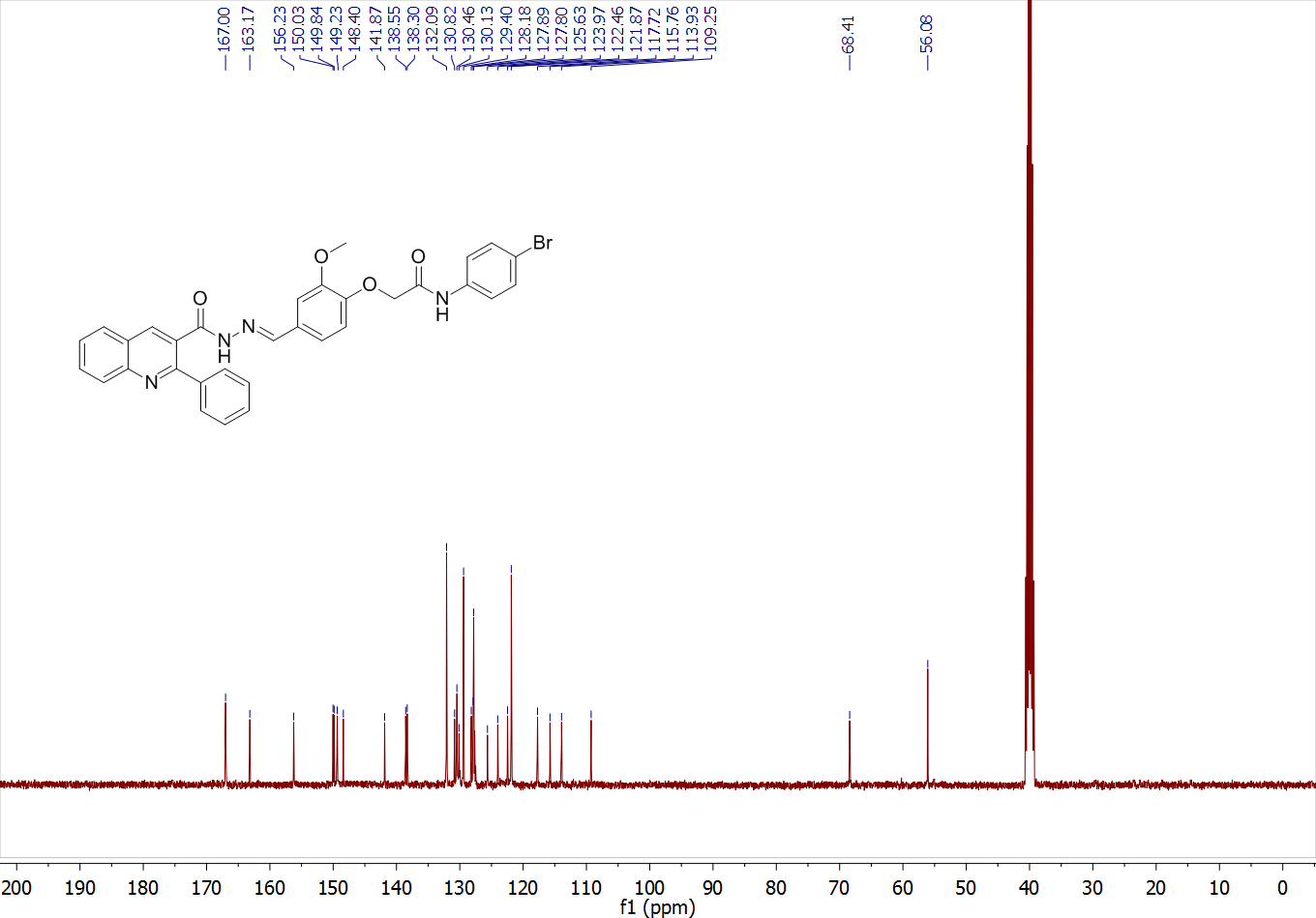


11h: 2-(2-methoxy-4-((2-(2-(4-methoxyphenyl)quinoline-4-carbonyl)hydrazineylidene)methyl)phenoxy)-N-phenylacetamide:


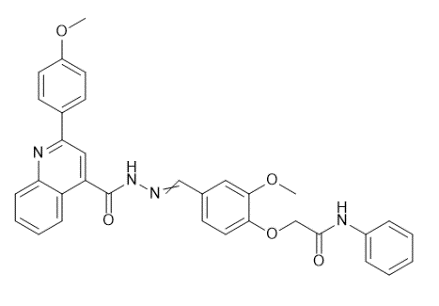

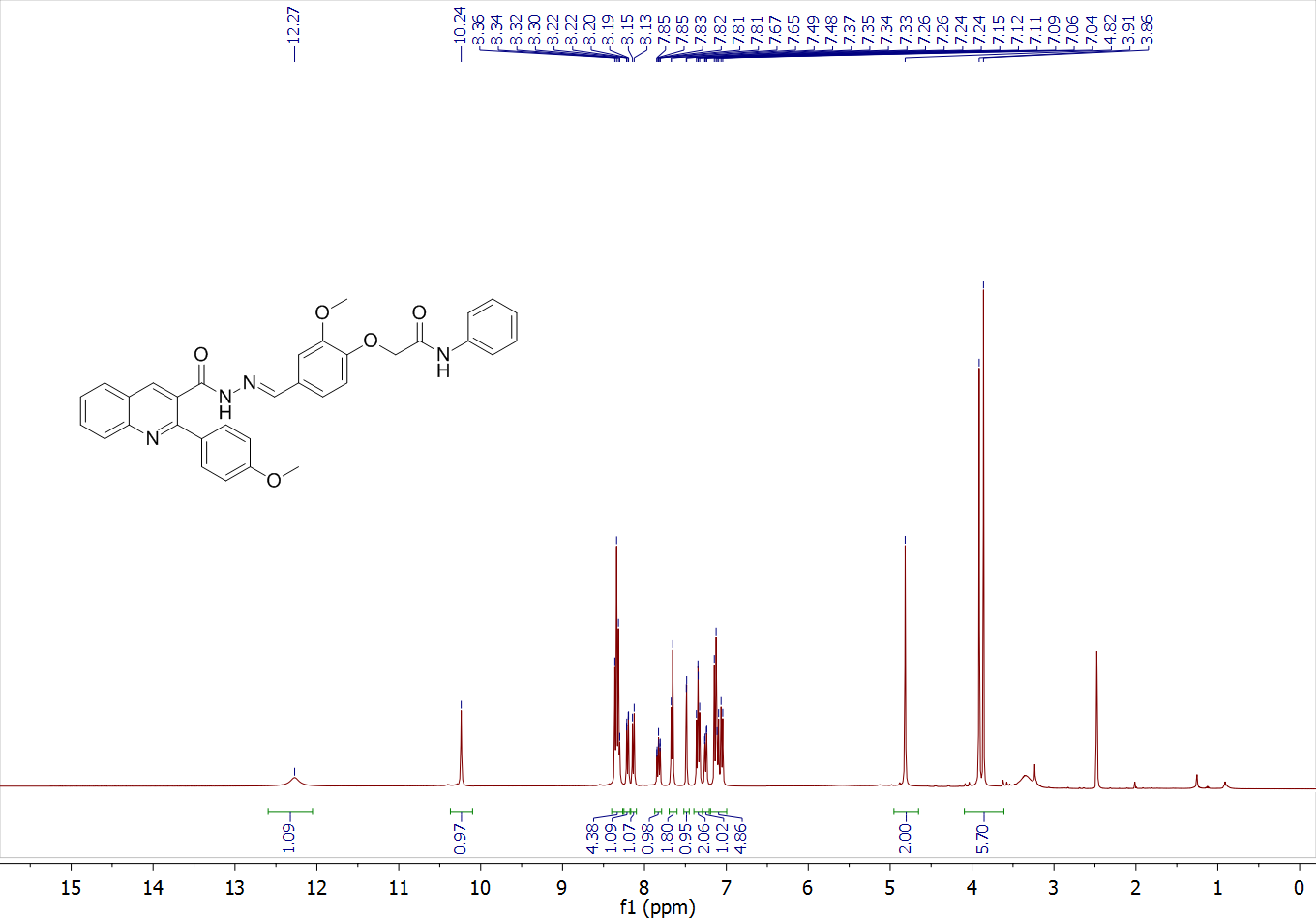


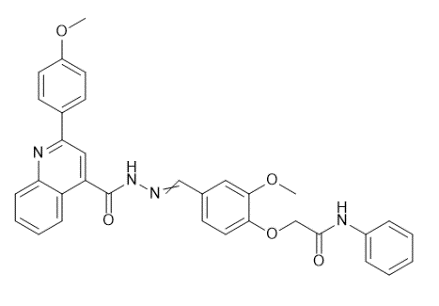
**
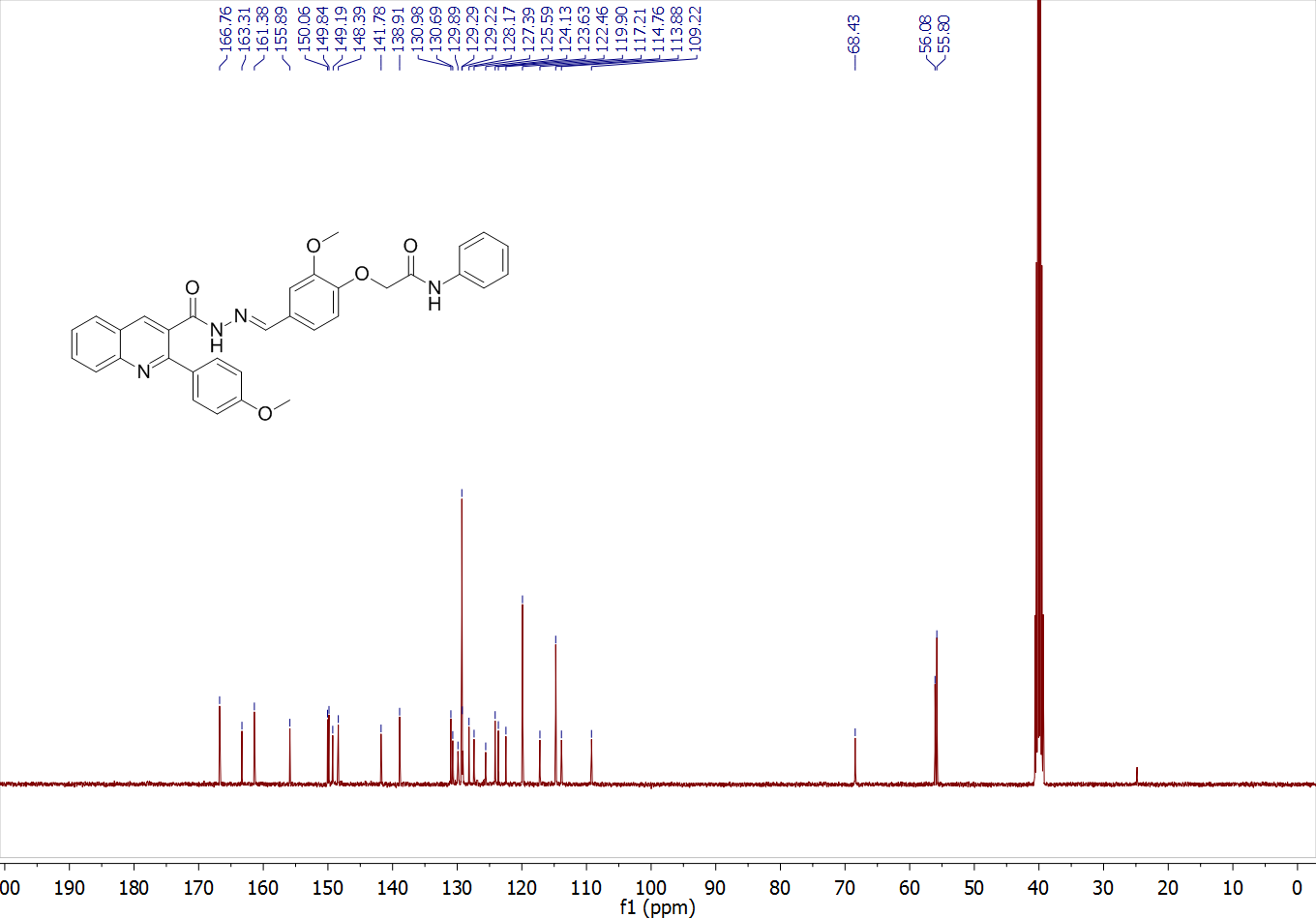
**

11i: 2-(2-methoxy-4-((2-(2-(4-methoxyphenyl)quinoline-4-carbonyl)hydrazineylidene)methyl)phenoxy)-N-(o-tolyl)acetamide:


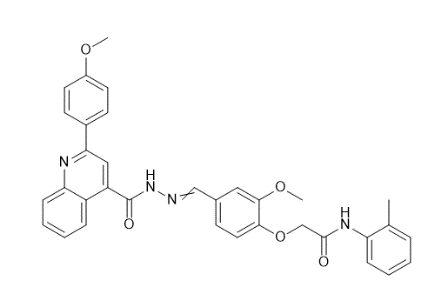

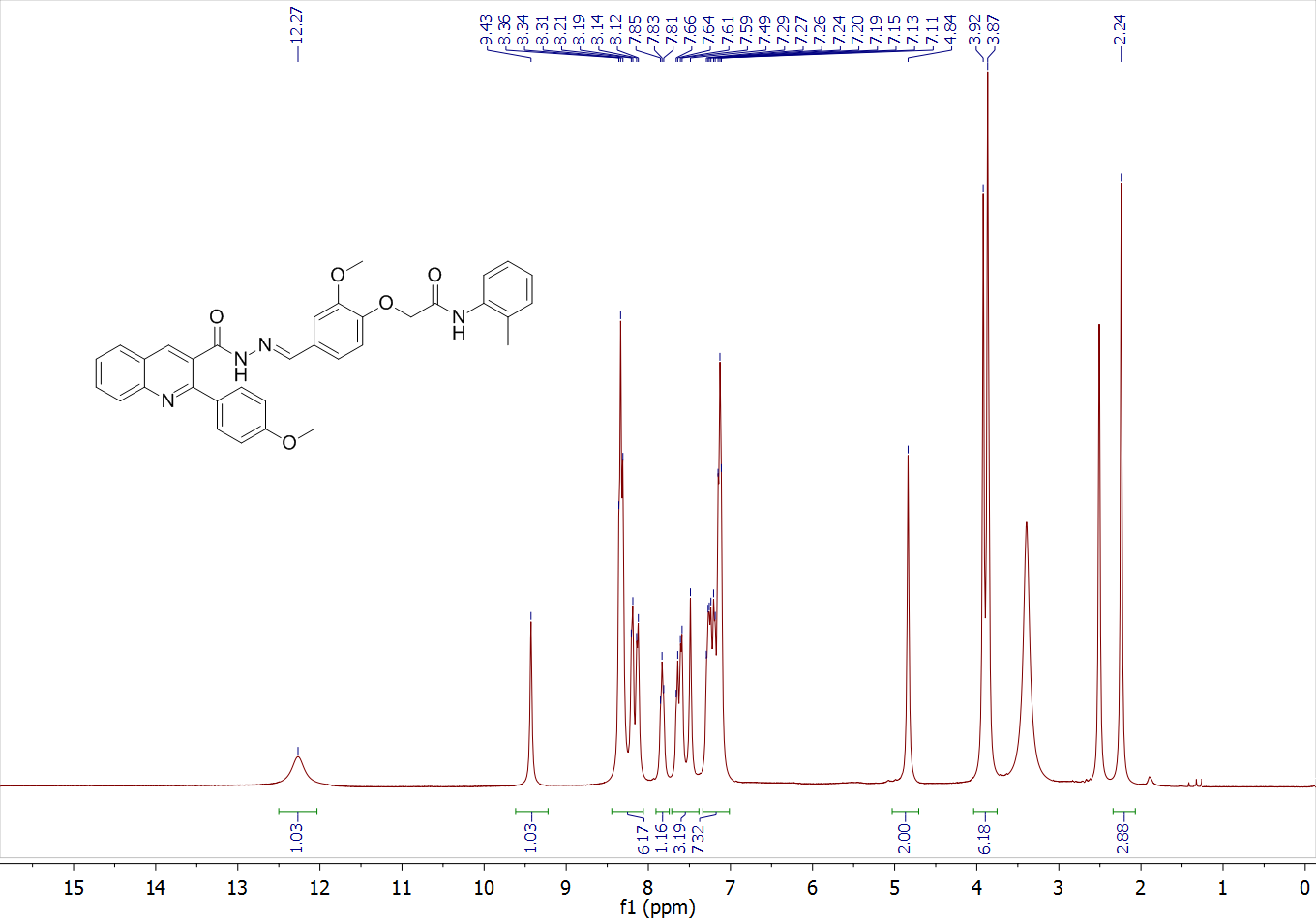


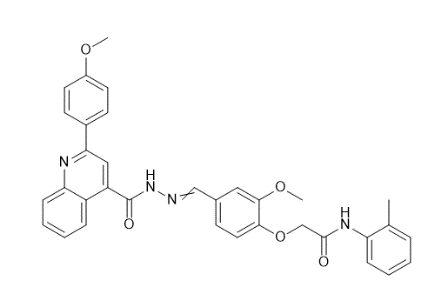

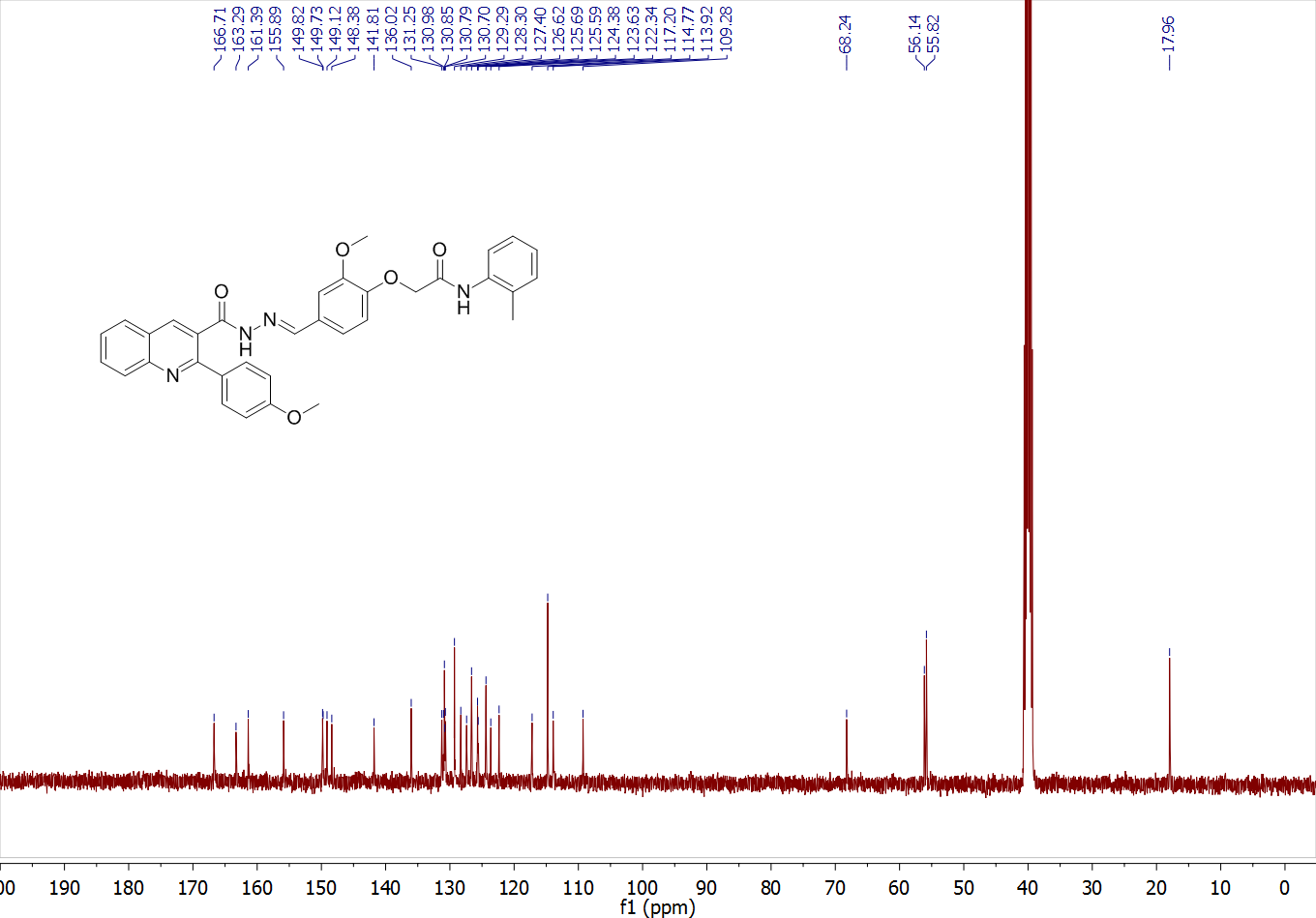


11j2-(2-methoxy-4-((2-(2-(4-methoxyphenyl)quinoline-4-carbonyl)hydrazineylidene)methyl)phenoxy)-N-(4-methoxyphenyl)acetamide:


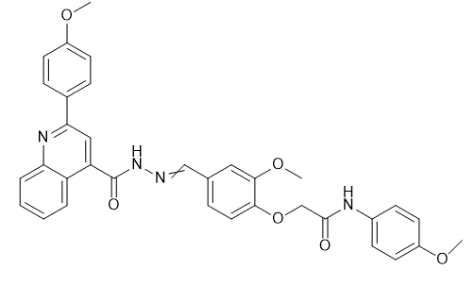

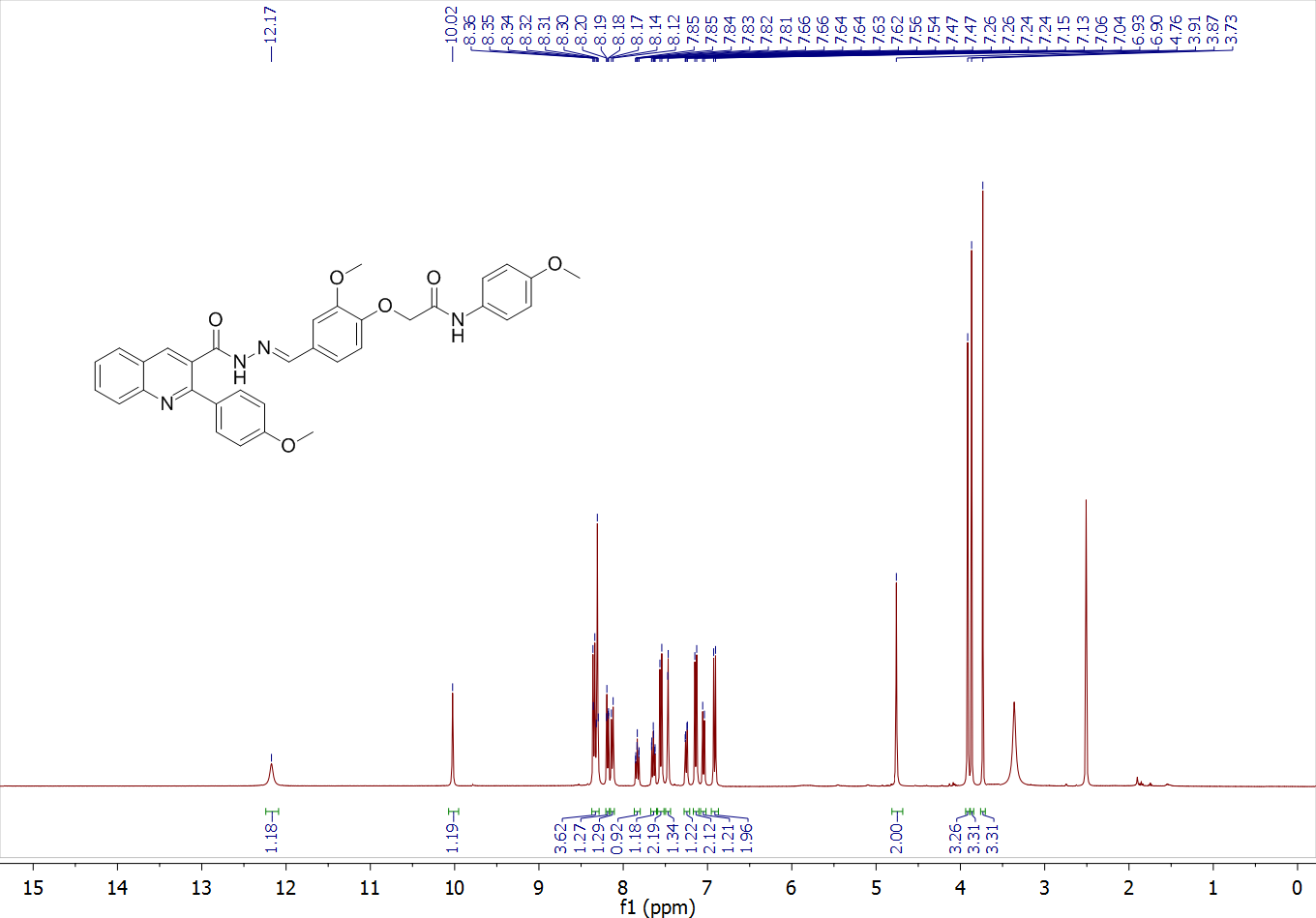


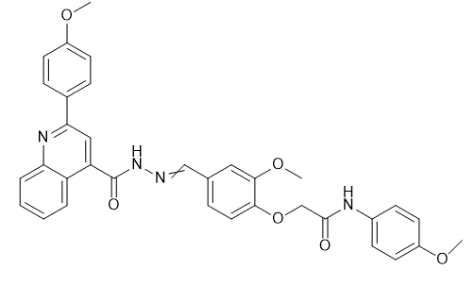

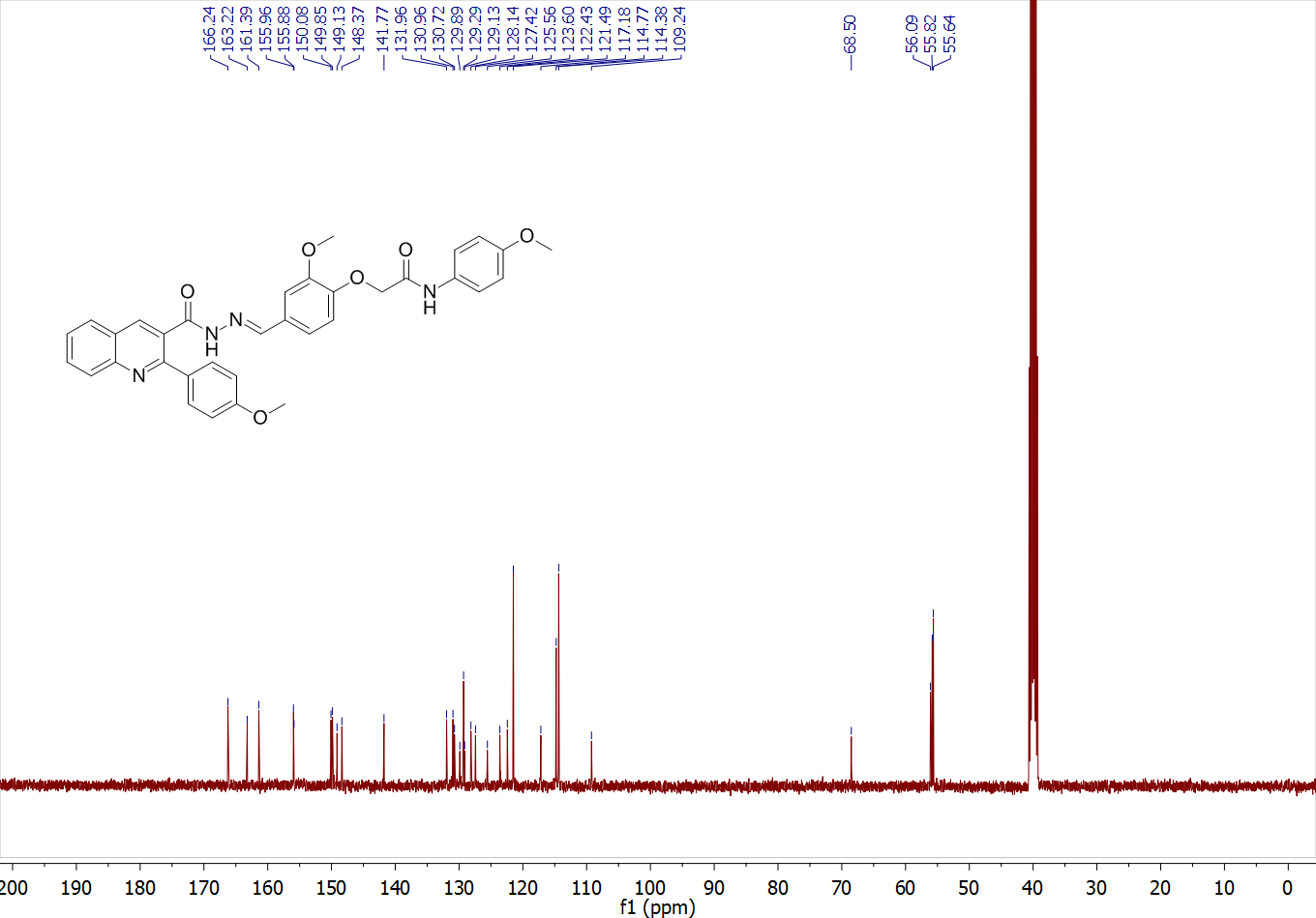


11k: N-(4-ethylphenyl)-2-(2-methoxy-4-((2-(2-(4-methoxyphenyl)quinoline-4-carbonyl)hydrazineylidene)methyl)phenoxy)acetamide:


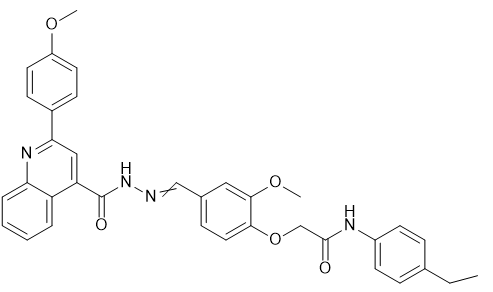

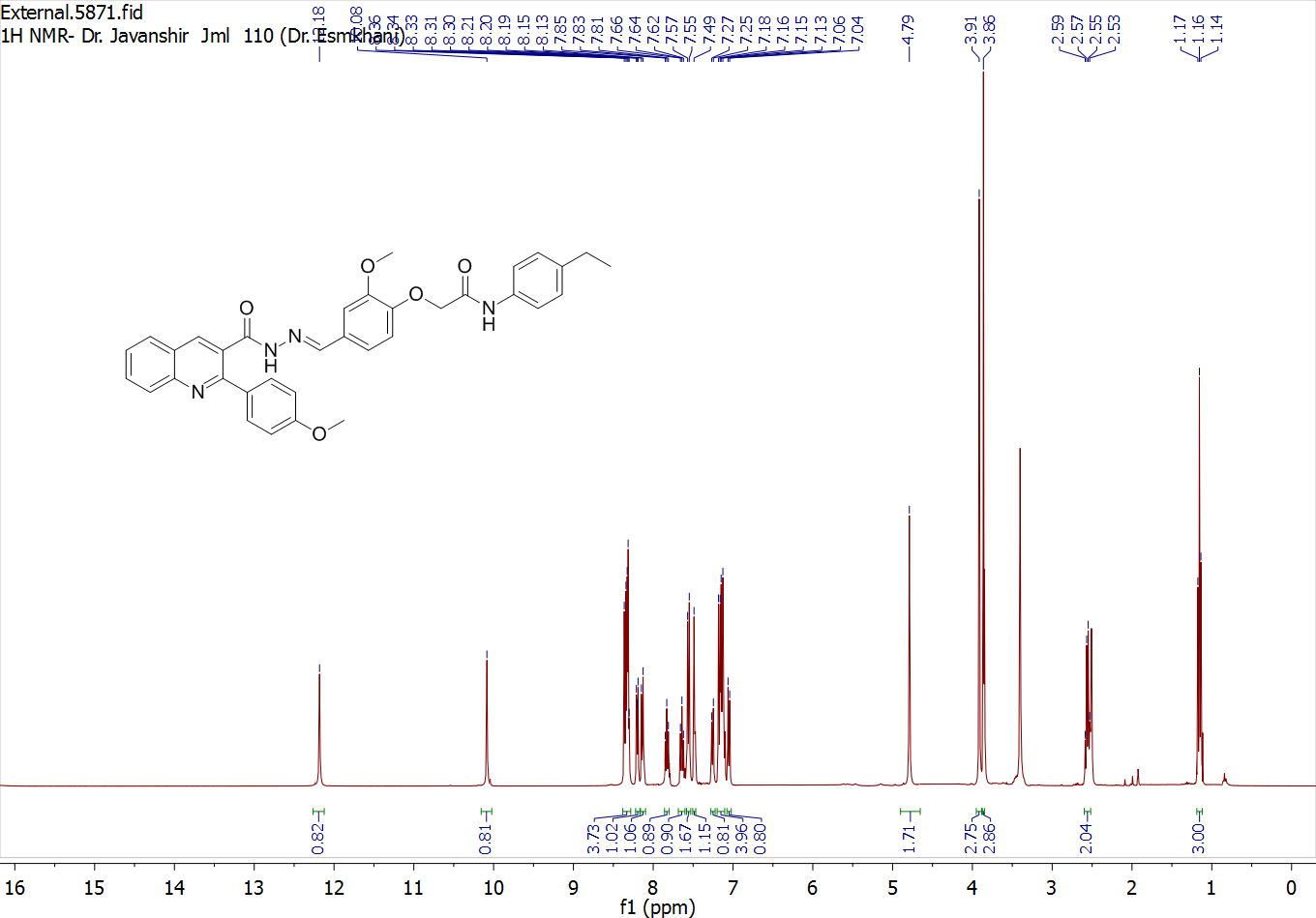


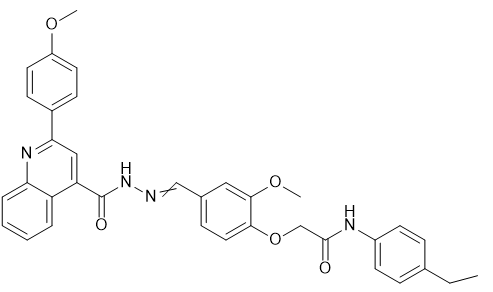

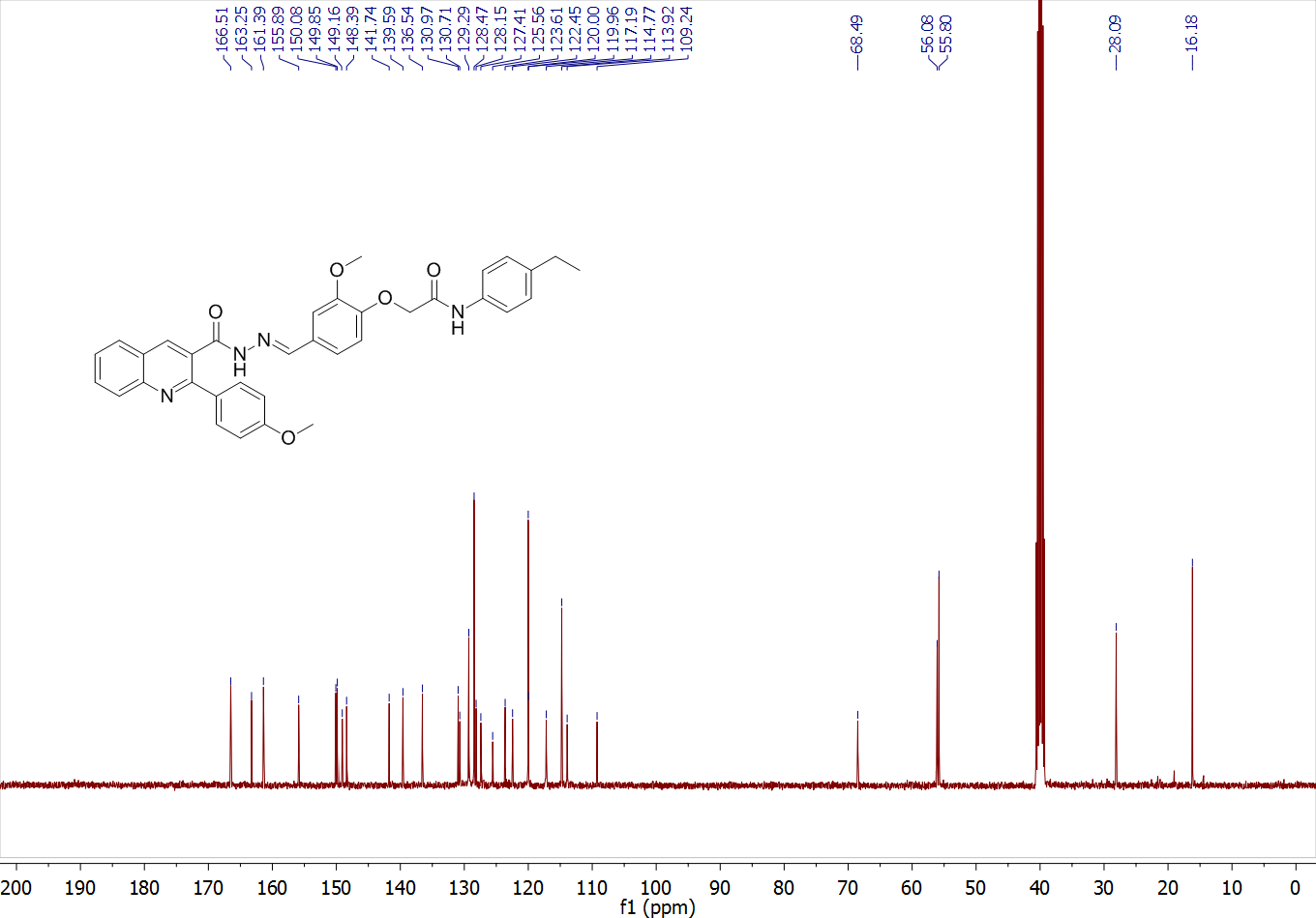


11l: N-(4-fluorophenyl)-2-(2-methoxy-4-((2-(2-(4-methoxyphenyl)quinoline-4-carbonyl)hydrazineylidene)methyl)phenoxy)acetamide:


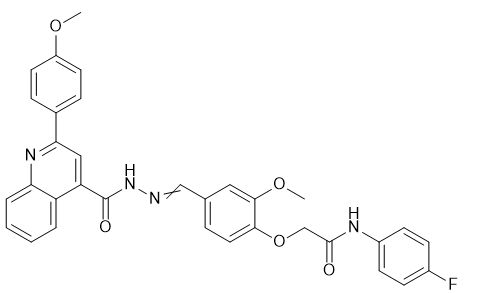

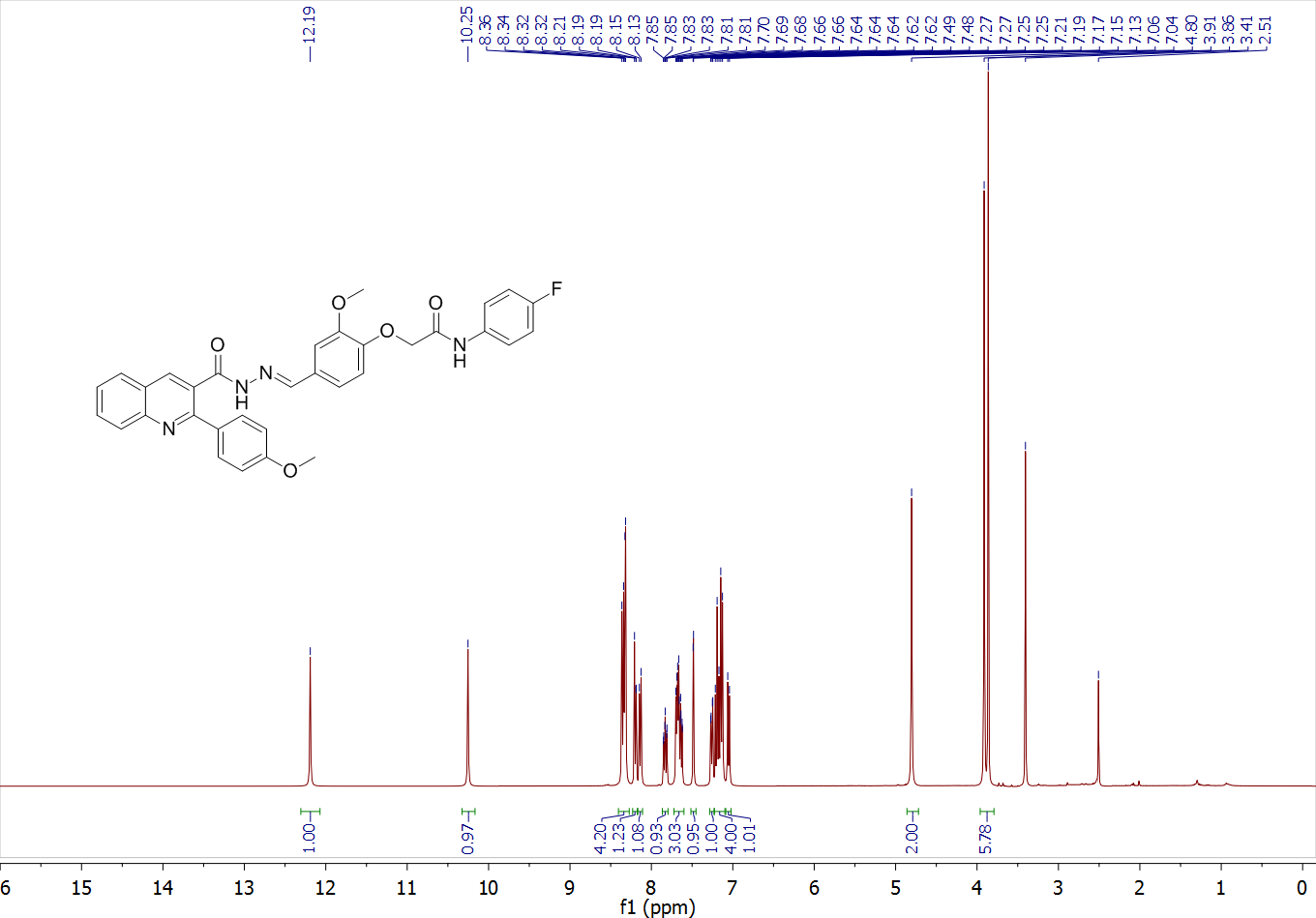


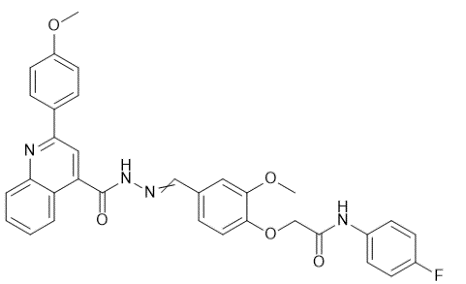

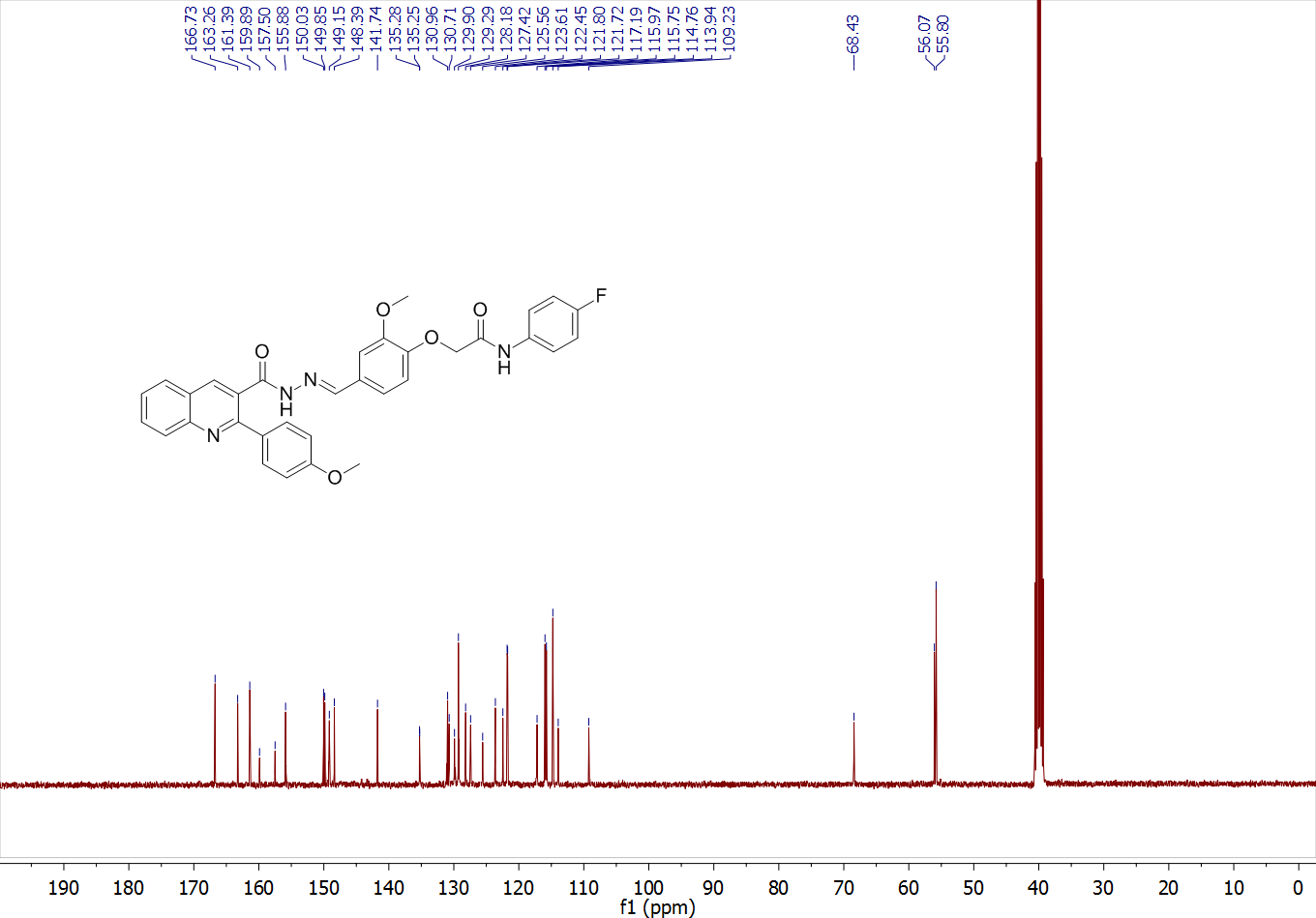


11m N-(4-bromophenyl)-2-(2-methoxy-4-((2-(2-(4-methoxyphenyl)quinoline-4-carbonyl)hydrazineylidene)methyl)phenoxy)acetamide:


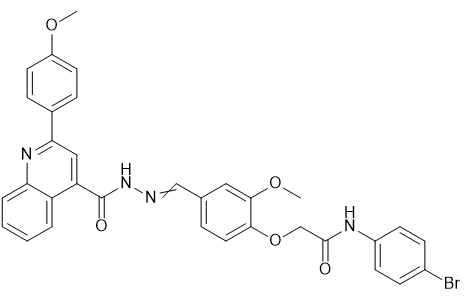

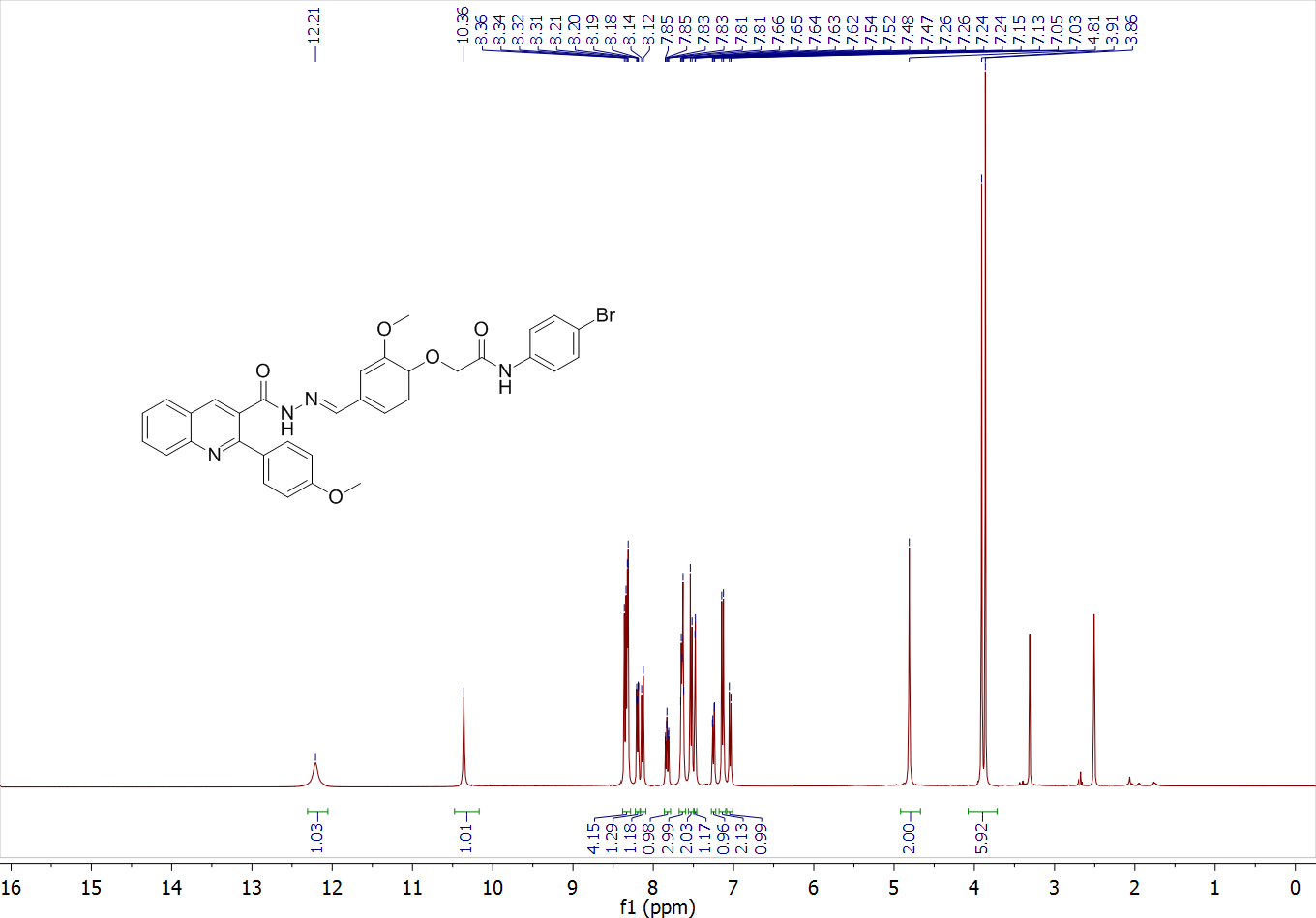


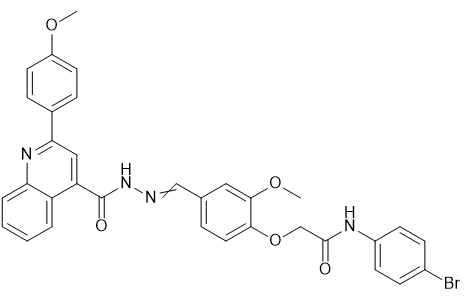

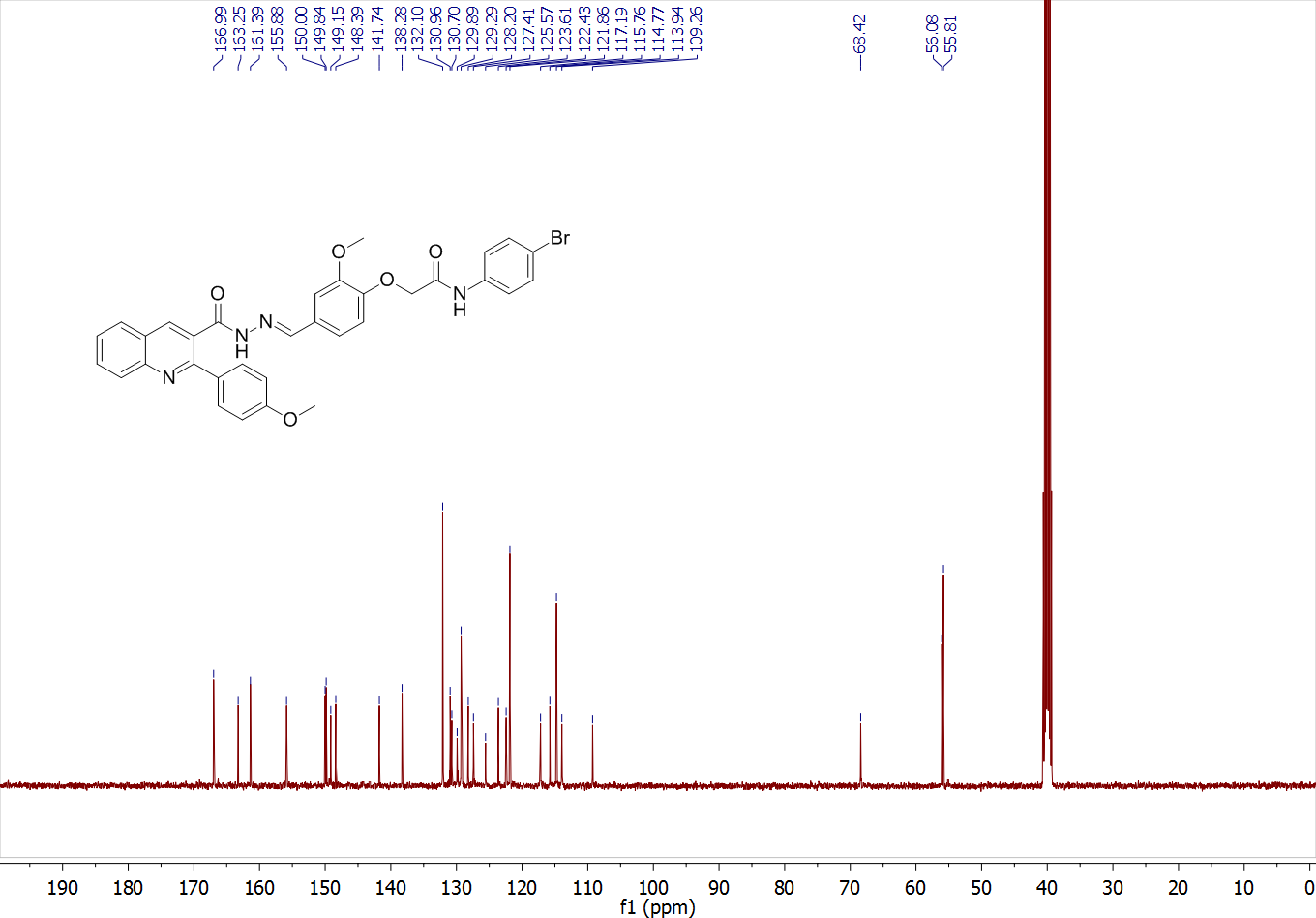


11n: N-(4-chlorophenyl)-2-(2-methoxy-4-((2-(2-(4-methoxyphenyl)quinoline-4-carbonyl)hydrazineylidene)methyl)phenoxy)acetamide:

:


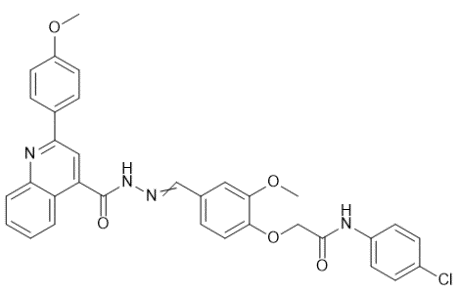

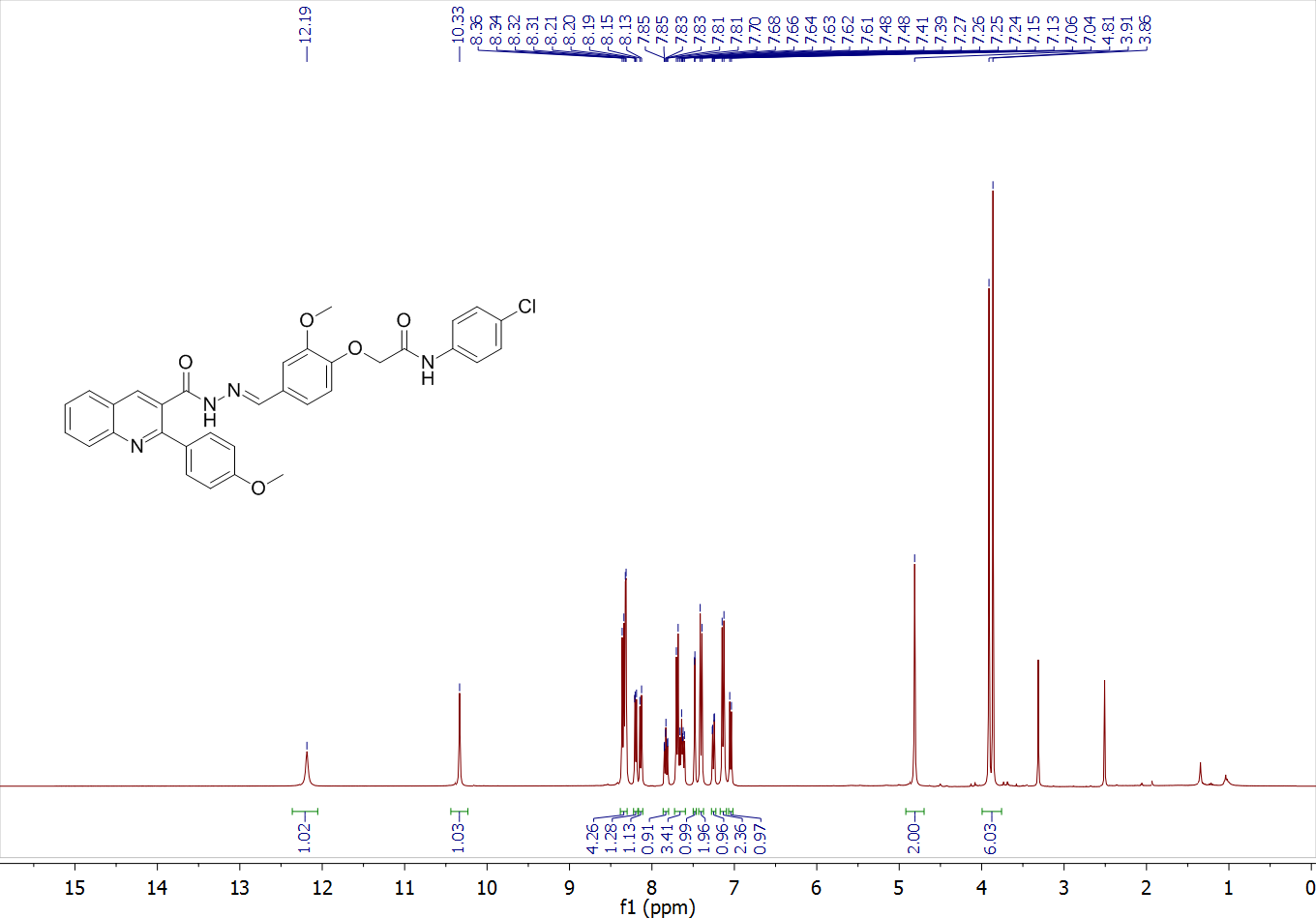


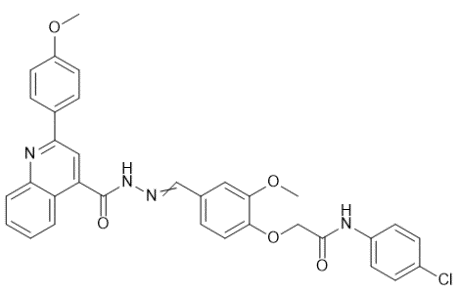

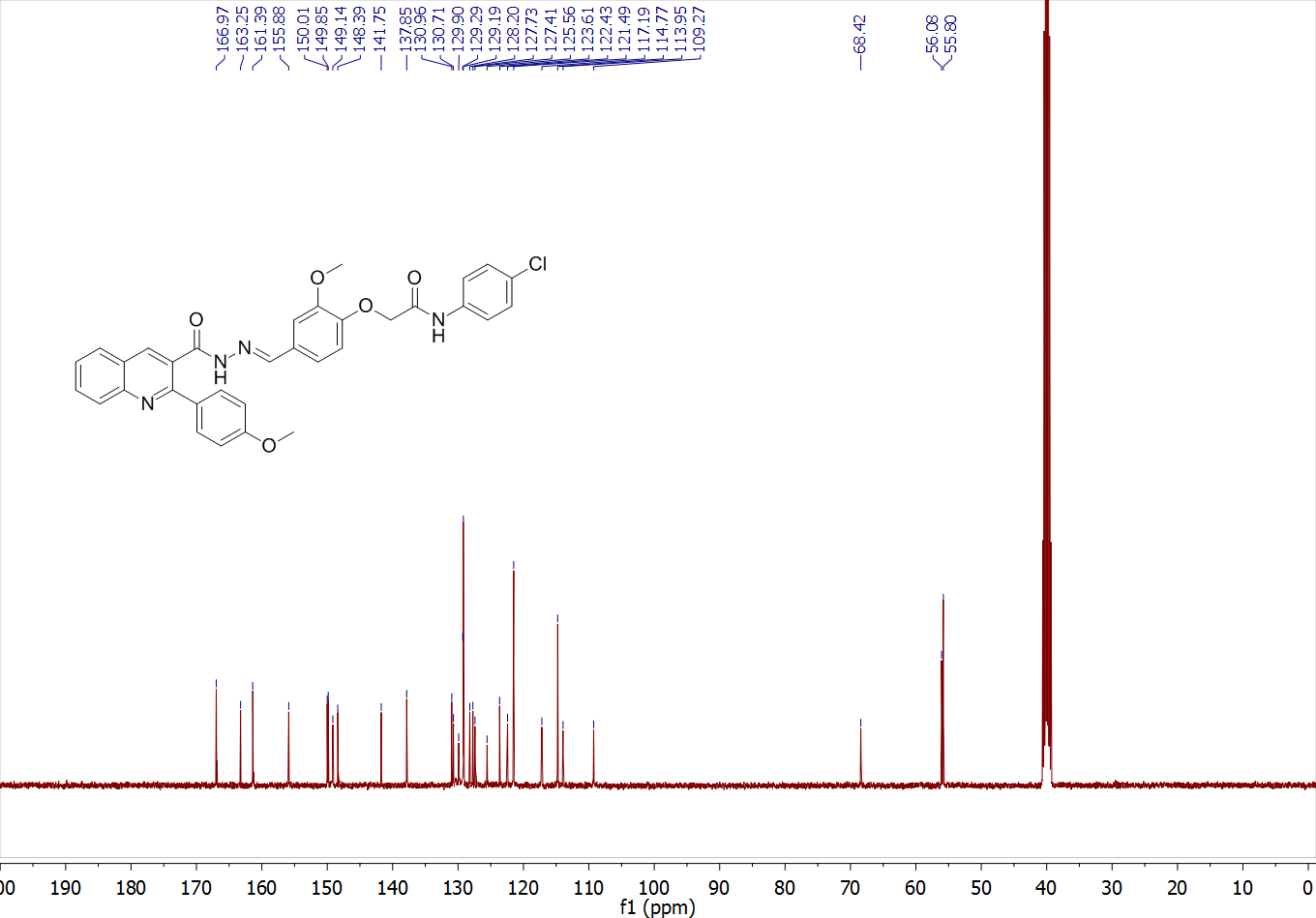


11o: 2-(2-methoxy-4-((2-(2-(4-methoxyphenyl)quinoline-4-carbonyl)hydrazineylidene)methyl)phenoxy)-N-(4-nitrophenyl)acetamide:


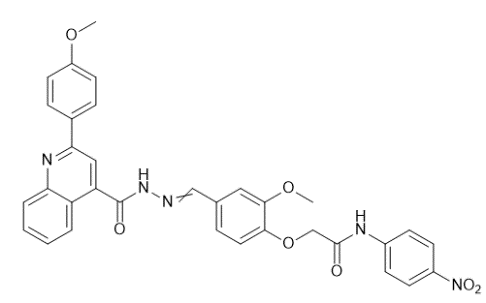

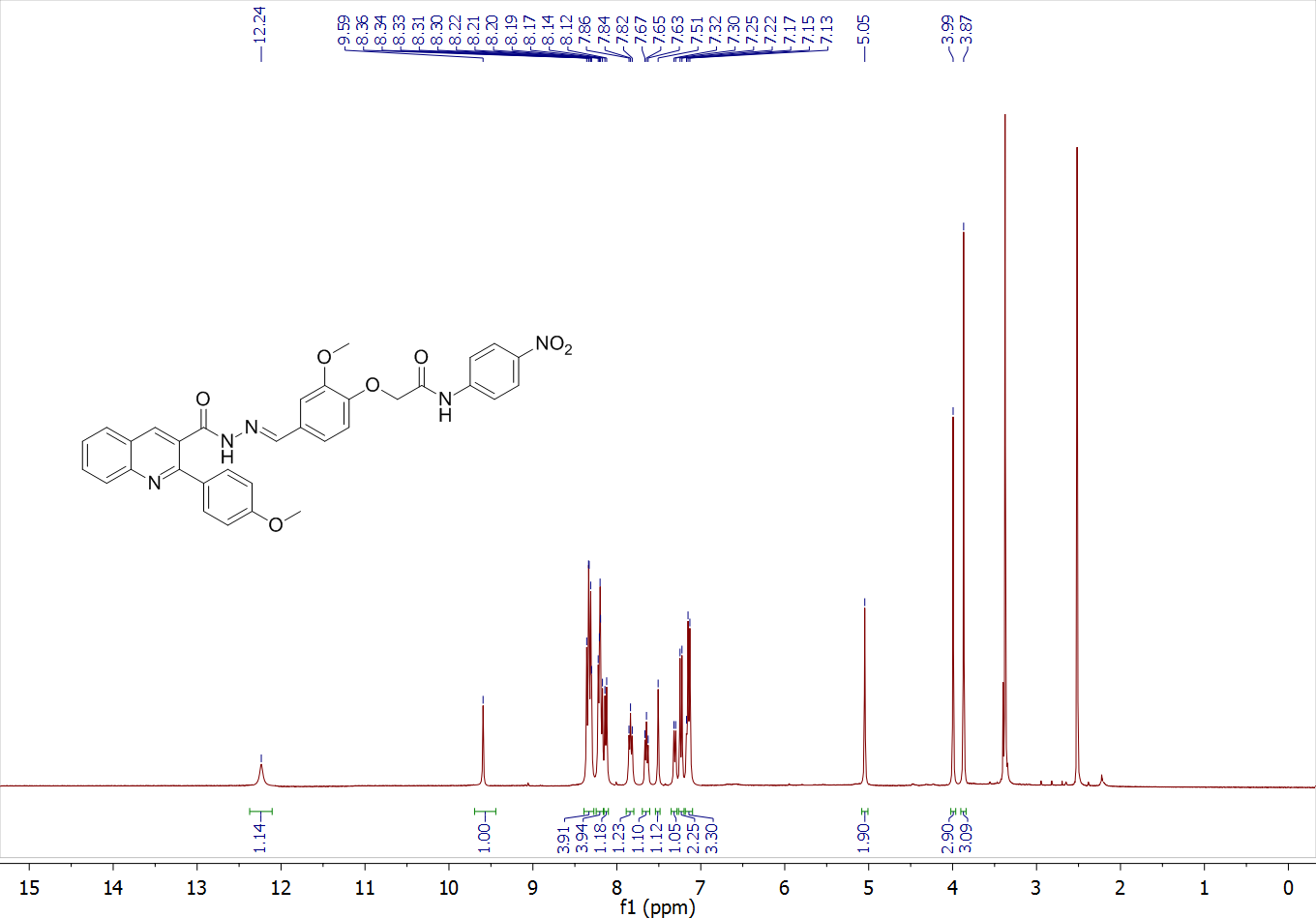


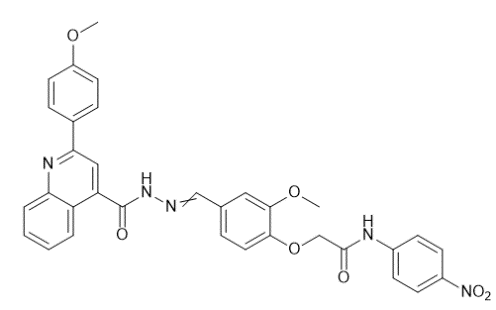

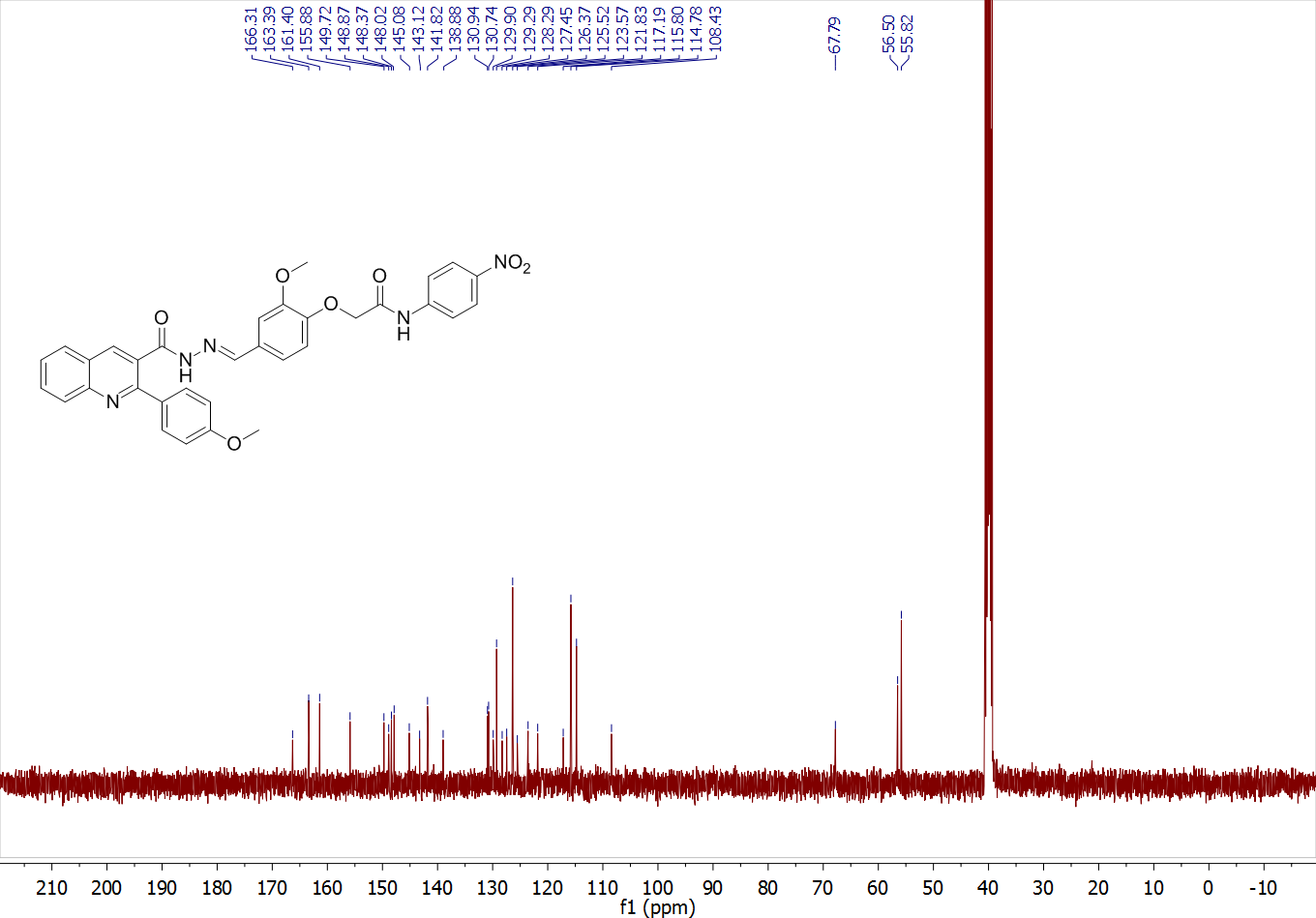

Supplement: Supplementary file 1 — Supplementary Information. [file 41598_2023_50395_MOESM1_ESM.docx]
